# Cellulose–Silver Composites Materials: Preparation and Applications

**DOI:** 10.3390/biom11111684

**Published:** 2021-11-12

**Authors:** Ahmed Salama, Ragab E. Abouzeid, Medhat E. Owda, Iriczalli Cruz-Maya, Vincenzo Guarino

**Affiliations:** 1 Cellulose and Paper Department, National Research Centre, 33 El-Bohouth St., Dokki, Giza 12622, Egypt; r_abouzeid2002@yahoo.com; 2 Chemistry Department, Faculty of Science, Al-Azhar University, Nasr City, Cairo 11884, Egypt; medhatowda@gmail.com; 3 Institute of Polymers, Composite and Biomaterials, National Research Council of Italy, Mostra D’Oltremare, Pad 20, V. J.F. Kennedy 54, 80125 Naples, Italy; cdiriczalli@gmail.com

**Keywords:** cellulose, silver, processing, composites, biomaterials

## Abstract

Cellulose has received great attention owing to its distinctive structural features, exciting physico−chemical properties, and varied applications. The combination of cellulose and silver nanoparticles currently allows to fabricate different promising functional nanocomposites with unique properties. The current work offers a wide and accurate overview of the preparation methods of cellulose–silver nanocomposite materials, also providing a punctual discussion of their potential applications in different fields (i.e., wound dressing, high-performance textiles, electronics, catalysis, sensing, antimicrobial filtering, and packaging). In particular, different preparation methods of cellulose/silver nanocomposites based on in situ thermal reduction, blending and dip-coating, or additive manufacturing techniques were thoroughly described. Hence, the correlations among the structure and physico–chemical properties in cellulose/silver nanocomposites were investigated in order to better control the final properties of the nanocomposites and analyze the key points and limitations of the current manufacturing approaches.

## 1. Introduction

Scientists are trying to develop novel materials derived from renewable resources and sustainable green materials to produce high-value products. During the last few decades, polysaccharide-based materials have been considered as promising candidates in terms of abundance of supply and environmental viability. Moreover, their use in various fields has grown quickly [1,2]. Polysaccharides have influenced most modern technologies and can be easily seen in several applications. Various materials, such as natural fibers, hydrogels, and polymer composites, have been developed by means of polysaccharides [3,4]. The unique properties of polysaccharides, such as high specific strength, low density, high thermal properties, and the availability from renewable feedstocks in large quantities, presented them as promising candidates. Moreover, polysaccharides are applied for tailoring unique materials to meet specific and desired industrial applications. These biomaterials have replaced synthetic polymers due to their sustainability, low cost, and biocompatibility [5]. Polysaccharides, such as cellulose, chitosan, starch, and alginate, are unique examples of environmentally friendly polymeric materials that are currently applied in several industrial and biomedical fields [3,6,7]. Many efforts have been performed to combine polysaccharides with other polymers to develop new products with improved eco-friendly properties [8]. Among them, cellulose and its derivatives have been applied to prepare high performance composites. The incorporation of cellulose results in new materials with specific advantages and unique properties. Many trials were carried out for using cellulose as a potential component for various polymer composite applications, e.g., drug delivery [9], wound dressing, coatings, superabsorbent hydrogels [10], and adsorbents [11,12], in place of the commercially used ones. The preparation of novel materials from cellulose and its derivatives will reduce the cost of polymer composite materials as well as possibly address the problem of its waste disposal. During the middle of the nineteenth century, a metal ion−cellulose complex was prepared through wood treatment with different metal salt solutions for timber preservation [13]. Recently, metal-ion-modified cellulose has been a matter of excessive attention in the detection, photo up-conversion, adsorption, catalysis, and biomaterials [14,15].

Several review articles have been published on cellulose-based inorganics, such as cellulose/gold and cellulose/calcium phosphate composites [16,17]. However, cellulose and silver composites are not carefully described in the recent literature. Therefore, the present article aims to review the feasibility of using cellulose to prepare novel cellulose/silver composites and reviews the recent developments in cellulose/silver composites. First, the common preparation techniques were discussed, then their potential applications in various fields, such as wound dressing, antibacterial reagents, water treatment, catalysis, and biosensors, were described. Finally, the review will conclude with the recent developments and future trends of cellulose/silver composites.

## 2. Cellulose Forms

Cellulose, the most abundant polysaccharide material on Earth, has excellent physico–chemical properties, such as low cost, abundant availability, rich surface chemistry, biodegradability, biocompatibility, high thermal stability, good mechanical properties, and environmental benignity. These unique properties allow it to be used in a wide variety of applications in textile, packaging, paper, and lightweight composites [1]. Various cellulosic materials, such as nanoporous cellulose gel [18], cellulose microfibrils [19], and bacterial cellulose [20], were applied as an assistant medium, either as reductants or stabilizers of silver nanoparticles. Due to the presence of hydrophilic hydroxyl groups and active sites, modified cellulose can interact with several inorganic fillers, metals, and oxides to endow more unique attributes and enrich the functional applications. New applications of cellulose nanocomposites have recently been developed, such as antibacterial therapy, catalysis, absorption, and biological analysis, along with other applications of cellulose for organic dyes removal [21] or heavy metals [22] and support for inorganic mineralization due to the availability for surface adjustment [16,23]. The abundance of hydroxyl groups in cellulose fibers can be used to modify the cellulose surface. The generated new groups can assist the formation and stabilization of new composites. Recently, the incorporation of inorganic materials, such as silica, calcium phosphate, zinc oxide, magnetite, and silver nanoparticles, into cellulose materials is suggested as a common method to improve its chemical properties for biomedical and environmental applications [24]. However, the hydrogen bonding networks are densely organized and packed, resulting in a strong water insoluble fiber with a high tolerance to most organic solvents [25].

Cellulose is the main component of vegetable fibers surrounded by lignin and hemicellulose. In the plant cell wall, cellulose chains are arranged into fibers with diameters of few nanometers that are aggregateduntil to form -fiber bundles or fibrils with micrometric size. The arrangement of the microfibrils is shown in Figure S1a. In the last few decades, nanotechnology related to cellulosic materials have generated great families, which are called nanocellulose [26]. In the literature, there is a wide variety of cellulose reported depending on the natural source and the isolation methods that present different properties, such as morphology, structure, and diameters. This review will be focused on nanocellulose as cellulose nanocrystals (CNC), cellulose nanofibers (CNF), and bacterial cellulose (BC), as shown in Figure S1 [27,28].

Cellulose nanofibers (CNFs) are obtained by mechanical shearing with or without chemical treatment for producing diameters less than 100 nm, and this is clear from Figure S2 (A and B). While the highly crystalline CNC of nanometers in length is obtained by using strong acid hydrolysis to soften and break down the less crystalline regions (Figure S2C), BC is prepared via incubating bacteria cultures for some days in aqueous media containing different carbon sources (Figure S2D). However, the nanocellulose acts as a breeding ground for bacteria because of its hygroscopic properties [29]. Therefore, cellulosic materials should have antibacterial and antifungal activity to resist the infection. The incorporation of antimicrobial nanoparticles into cellulose can reduce negative effects associated with allergy and reproduction. Moreover, the shapes and sizes of the fibers can be modified, and the oxidized cellulose mediated by TEMPO (2,2,6,6-tetramethylpiperidine-1-oxyl) and sodium periodate can form cellulosic bundles with active sites (carboxylic and aldehyde groups) linking to antibacterial metal nanoparticles in various ways [30].

Cellulose-based antimicrobial materials can be broadly defined as a mixture of cellulose and antimicrobials that give new properties to this mixture and expand the applications of cellulose materials. Usually, there are three types of antimicrobials: (1) organic antimicrobials, such as lipids, alcohols, and phenols; (2) natural biological antimicrobials, which include amino acids, natural peptides, and polysaccharides; and (3) inorganic antimicrobials, such as silver, zinc, copper, etc., which have high antimicrobial activity and low toxicity. Silver nanoparticles (AgNPs) have become important antimicrobials, which have been shown to possess effective antibacterial activity through mechanisms that include the release of Ag^+^ ions affecting DNA replication or the breakdown of the proton driving force across the cell membrane, which has become a major target. The benefit is that it is relatively non-toxic to human cells. Cellulose compounds appeared as aids in the assembly and stabilization of metal oxide nanoparticles. It has been reported that cellulose assists metal oxides to be more stable with good performance and, consequently, widen the application of metal oxides for different applications. The cellulose fibers hold plentiful surface OH groups and essential porous structures, which suggests them as models to construct the cellulose/silver composites. These cellulose/silver composites combine the processability of cellulose fibers and the greater performances of silver materials. The homogeneous dispersion of silver nanoparticles over the cellulose fibers is a challenge during the fabrication process due to the aggregation of silver nanomaterials. This aggregation process was found to reduce the performances of the obtained composites. To reduce this problem, many techniques have been established to prepare or immobilize silver nanoparticles on cellulose, such as hydrolysis and the sonochemistry technique [31]. Metal oxides could be incorporated into cellulose through a one- or two-step process. The most suitable technique is the formation of monodisperse nanoparticles followed by assembling these nanoparticles onto cellulose fibers. This technique assists the formation of nanoparticles with a uniform size and morphology [32]. The bonding between cellulosic materials and silver nanoparticles takes place in three types of bonding to form antimicrobial materials, such as physisorption, electrostatic binding, and charge transfer reactions. Among the preparation techniques, the in situ formation shows several advantages due to the use of simple reaction procedures, light reaction conditions, and higher yield. During the last few years, many trials have been applied to fabricate silver-nanoparticles-decorated functionalized cellulose nanofibers. Promising results on cellulose/silver nanocomposites based on silver-decorated nano-nanohybrids have succeeded in constructing a controlled release system and a long-acting antibacterial system. The aldehyde and carboxylic groups were used as reducing agents and stabilizers to assist the silver nanoparticles formation.

### 2.1. Cellulose Derivatives/Silver Nanocomposites

Recently, several experiments have been developed to fabricate cellulose/AgNPs nanocomposites. For example, the in situ synthesis of AgNPs in cellulose fibers in the presence of sodium borohydride as a reducing agent was studied by Zhu et al., 2009. It was found that the sizes of the formed AgNPs change with the change of NaBH_4_ concentrations [33]. The oxidation of cellulose surfaces with a periodate was carried out to generate two aldehyde groups per glucopyranose unit. The formed dialdehydes were examined for the precipitation of silver particles by the Tollens (silver mirror) reaction. The formed composites were a semiconductor, with σ ≈ 5 S/cm. These compounds are possible to be valuable in electronic, chemical sensing, and catalytic materials [34]. Dicarboxylic cellulose was prepared through the oxidation of cellulose by oxidizing the neighboring hydroxyl groups at positions C2 and C3 with NaIO_4_. The modified cellulose assisted the deposition of uniform silver nanoparticles decorated with approximately 15 nm size.

Dicarboxylate cellulose/silver nanocomposite showed excellent antibacterial activity against gram-positive and gram-negative bacteria [19]. Y. Wang prepared multifunctional composite films by using isophorone diisocyanate as a coupling agent between the OH and COOH groups of graphene oxide and the OH groups of silver-coated zinc oxide nanoparticles into the hydroxypropyl cellulose. The nanocomposite film prepared by solution blending showed an improved mechanical performance and strong UV resistance. These can effectively deactivate both gram-negative bacteria (*E. coli*) and gram-positive bacteria (*S. aureus*) and have also been shown to accelerate wound healing and antibacterial properties [35]. The non-toxic green reducing agents, such as ethylene glycol (EG), glucose, and ascorbic acid, are commonly chosen for the synthesis of cellulose/Ag nanocomposites using a microwave-assisted method. Organic solvents and toxic chemicals were replaced during the preparation of the AgNPs nanocomposites because they are harmful to the environment. This greatly limits the actual application in biomedical fields. However, due to the weak reduction of these reducing agents, larger sizes of particles or clusters of AgNPs were observed in cellulose, resulting in weak antibacterial effects [36,37,38].

Ascorbic acid was used as a reducing agent for the silver for preparing silver particles with 250 nm, which is relatively weakly diffused in a matrix of cellulose [37]. In order to additionally improve the greenness of the in situ era of the AgNPs, utilizing a few natural substances, such as counting plant and plant extract, was investigated for preparing silver nanoparticles. Aladpoosh et al. fabricated AgNPs/cotton fibers by using *S. rosmarinus* ash as a reducing agent, with the accumulation of silver particles in the matrix being unacceptable [39].

Cellulose derivatives, such as methyl cellulose (MC), hydroxyethylcellulose (HEC), and hydroxypropyl methylcellulose (HPMC), were used as reducing agents for the precipitation of silver nanoparticles (AgNPs) and compared for their reducing properties. HPMC presented the highest reducing power, with an equilibrium concentration (EC) of 84.6 ± 4.5 μmol Fe^2+^/g, followed by MC and HEC, with an EC of 62.3 ± 1.4 and 38.1 ± 3.2 μmol Fe^2+^/g, respectively [40].

There are several methods to prepare the mixture of cellulose with silver nanoparticles, such as in situ chemical reduction and covalent bonding methods [41]. The physical method includes the use of cellulose and silver nanoparticles, which were prepared separately followed by mixing without any chemical reactions. In this case, cellulose materials act as a carrier (Figure S3). This method is a very simple process with a relatively high silver loading ratio. Due to the relatively weak bonding between cellulose and silver, the particles adsorbed on the surface of materials may be lost during use. In other words, products prepared in this way have poor washing durability.

**Figure 1:**
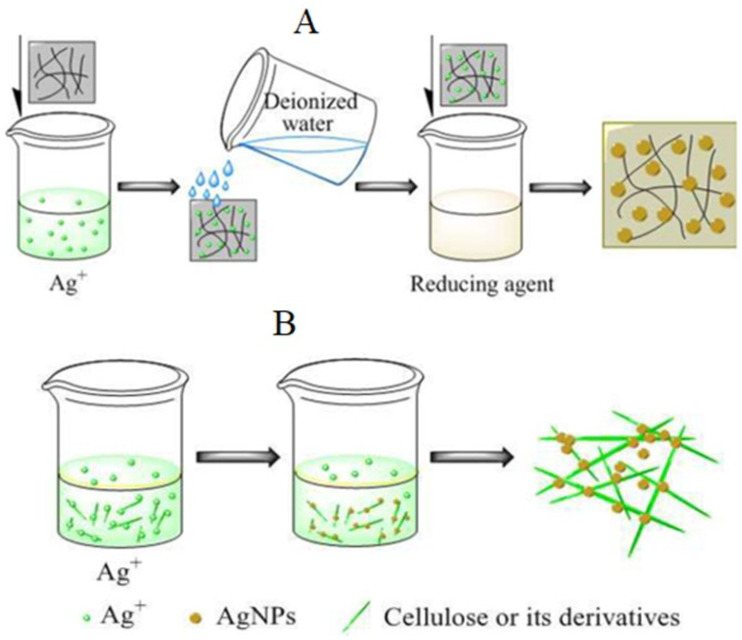
Chemical reduction methods of (AgNPs–cellulose composites). (**A**) Cellulosic materials act as both a matrix and a reducing agent; (**B**) cellulosic materials serve as a matrix [41].

In order to overcome the defects of the efficiency of the physical adsorption of the physical method, the in situ chemical reduction method was widely used. Compared with physical adsorption, in situ chemical reduction includes the slow growth of AgNPs with the formation of small diameters; in this case, cellulosic materials play a dual role (reducing agent and matrix), as shown in Figure 1.

### 2.2. Cellulose Nanomaterials/Silver Nanocomposites

The acid treatment of cellulose degrades amorphous domains, leaving behind rod-like cellulose nanocrystals (CNCs) that have unique chemical and mechanical properties. Cellulose nanocrystals have a good probability as a host material due to their high specific surface area and high dispersibility. There are interesting trials for understanding the role of CNCs’ surface chemistry in silver nanoparticle synthesis. For example, the effect of the presence of CNCs on the nature of silver nanoparticles designed by the chemical reduction of silver ions using sodium borohydride was studied [42]. Cellulose nanocrystals were applied to facilitate silver nanoparticle synthesis through the assistance of nanoparticle formation and nucleation in the presence of reducing agents for producing silver nanoparticles of different sizes and different rates of formation. The percentage of small silver nanoparticles (from 1 to 5 nm) was increased with increasing the concentrations of both untreated and desulfated CNCs, which confirms the role of CNs to minimize the growth of silver nanoparticles through steric hindrance. The small silver nanoparticles and the increased rate of the formation of nanoparticles with the increase of the concentration of nanocrystals indicates the nucleation controlling capability of CNs. The authors concluded that CNCs in their modified or unmodified states have a high potential for nucleation controlling and nanoparticle stabilizing properties [43].

The optimization of the density of the silver nanoparticles on the surface of the CNCs was carried out through controlling the size and concentration of the suspension. For example, the pulsed synthesis process produced size-limited silver nanoparticles with a maximum average particle size of 32 nm [44]. Nanocrystalline cellulose/silver nanoparticles composites were prepared by a liquid phase chemical reduction technique using glucose as a reducing agent. The formed silver nanoparticles exhibited a diameter of ~3 to 6 nm, and a content of 8 wt% was homogenously complexed onto the fiber surfaces [45]. Li Fan and Hui Zhang et al. studied the use of CNCs as carriers and stabilizers for AgNPs on polyvinyl chloride nanocomposites (Figure S4). It was found that the AgNPs have been successfully prepared with an average size of 25 nm in spherical form and dispersed uniformly on the surface of CN-like uniform sizes [46].

**Figure 2:**
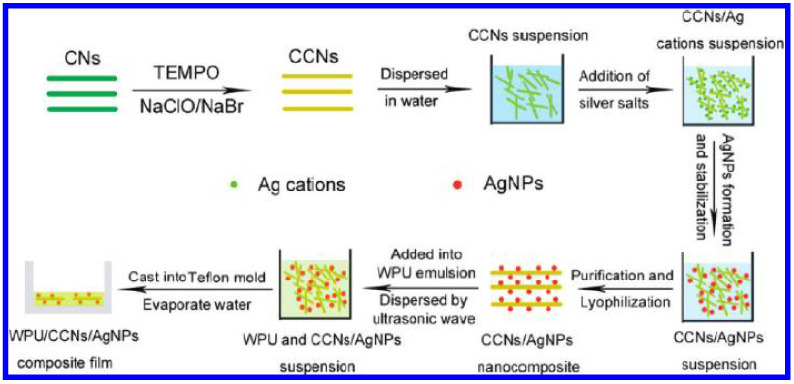
Preparing the CCNs/AgNP composite and incorporation into waterborne polyurethane [47]. Copyright © 2012, American Chemical Society.

The development of bio-compounds from renewable biomass complements viable materials produced from mineral fuels and fossil resources. Compounds consisting of cellulose nanocrystals (CNs) to prepare carboxylated cellulose nanocrystals (CCNs) and silver nanoparticles (CCNs/AgNP) were applied as a dual function to develop the mechanical and antimicrobial properties of waterborne polyurethane, as shown in Figure 2 [47].

### 2.3. Bacterial Cellulose/Silver Nanocomposites

**Figure 3:**
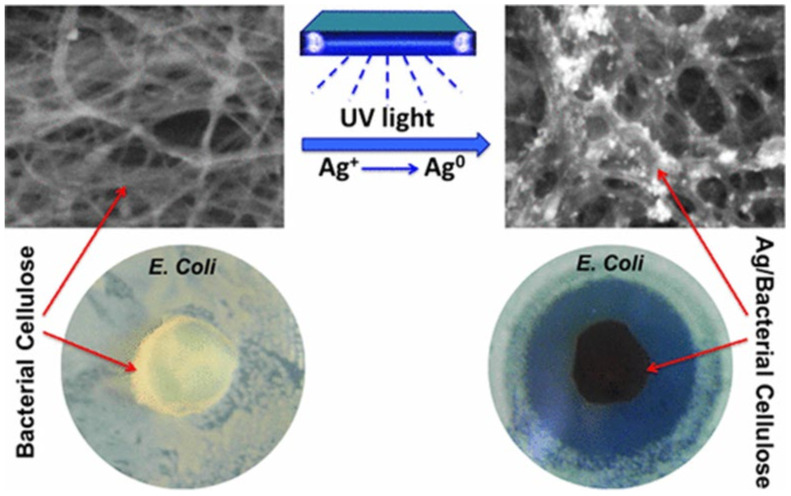
Formation of silver nanoparticles on the BC using UV light and its antimicrobial activity [20].

Bacterial cellulose was synthesized for the first time from the extracellular matrix of *Acetobacter xylinum* by Brown in 1886 [48]. Bacteria cellulose (BC) is formed by the fermentation of gram-negative bacterium *Acetobacter xylinum*, which can yield a unique 3D web-like nanofibrous network structure [49,50]. The produced nanofibers have high water holding capacity, high mechanical strength, and formability, especially in wet form. They are produced as an ultrafine network with a gel-like composition showing high potential in regeneration and wound healing applications [51]. Moreover, they could be prepared with high purity without harsh chemical treatment [52]. The presence of a high density of hydroxyl groups could improve the ability for the functionalization with different kinds of nanoparticles. BC was introduced as a natural wound dressing material thanks to the high porosity of the formed mesh and a higher water holding capacity. However, BC’s antimicrobial deficiency hampered its application in biomedical applications. Bacterial cellulose that consists of nanofibers nearly 10 nm thick and 50 nm has a high Young’s modulus ~138 GPa and tensile strength higher than 2 GPa. The use of bacterial cellulose as a wound dressing with some functional components can reduce wound infection and improves the delivery of medicines to the infected site [53]. Silver nanoparticles were deposited in the BC and synthesized by a UV photochemical reduction process. The preparation of a BC/Ag hybrid composite as an antibacterial membrane for wound healing applications was reported. For example, the Ag/BC hybrid nanocomposite formation under UV light irradiation from Ag-impregnated onto pure BC is illustrated in Figure 3 [20].

Therefore, bacterial cellulose has been presented as a good template for fabricating metallic nanoparticles with a high surface-to-volume ratio, high mechanical stiffness, and low thermal expansion properties [54]. However, preparing bacterial cellulose/metal nanoparticles through the electrostatic interactions of metallic ions and dipole moments of cellulose molecules produces a low yield of immobilized metallic ions on cellulose [55].

Bacterial cellulose has few downsides, for example non-water-solubility, low adsorption capacity, and variable physical stability. Different bacterial cellulose derivatives produced from the carboxymethylation, acetylation, and oxidation have been prepared to overcome these limitations. Kaisheng et al. prepared bacterial cellulose film containing silver sulfadiazine that was applied for in vivo wound dressing material. The histological results exhibited that bacterial cellulose/silver sulfadiazine film improves the epithelialization procedure in rats [56]. Several trials have been reported to enhance the interaction between guest metal ions and host cellulose fibers for preparing densely immobilized metal nanoparticles. The catalytic oxidation of cellulose by 2,2,6,6-tetramethylpyperidine-1-oxy radical (TEMPO) has been reported to generate carboxylate groups into the primary hydroxyl group. The TEMPO-mediated oxidation to BC can proceed under mild aqueous conditions. Moreover, the crystallinity and crystal size of the BC are unaffected after introducing the carboxyl group. The introduced carboxylate groups can act as a host for the introduced guest metal ions through an ion-exchange reaction [54]. For example, the ion exchange between sodium, from oxidized cellulose, and silver salt was carried out through the silver solution, followed by thermal reduction. This strategy created silver nanoparticles with a controlled size distribution with a high density through particle interactions between the host carboxylate groups and guest silver ions [54]. Bacterial cellulose was chemically modified with dopamine (catechol-containing compound) to give an adhesive nature to synthesize a mussel mimetic transdermal patch. The isolated bacterial cellulose was modified by the amidation reaction between carboxylate bacterial cellulose and dopamine (DOPA) for preparing DOPA-modified BC, which was applied for preparing a composite film containing reduced graphene oxide/silver nanoparticles. The composite film showed high antimicrobial action against both gram-positive and gram-negative bacteria. The cytotoxicity for the prepared films was investigated over the NIH3T3 fibroblast cell line. The results displayed that the Ag nanoparticles enhance the proliferation and the migration of the NIH3T3 fibroblast cells, as well as A549 human lung epithelial cells, which are responsible for improving wound healing [57]. Alonso-Díaz prepared environmentally friendly nanocomposites by an in situ thermal reduction under microwave to obtain silver nanoparticles anchored to the bacterial cellulose to avoid their release during applications and to increase their efficiency [58]. The nanocomposite showed positive results against the bacteria *E. coli* and *Pseudomonas* syringae and the fungus *Botritis cinerea*. The pathogen inhibitory properties of the material showed no defense response activation, probably due to an early neutralization of the pathogen on site by the nanocomposite. Moreover, the strong anchoring of the silver nanoparticles onto bacterial cellulose reduced their release and, consequently, suggests that the nanocomposite is potentially safer for the environment [58]. In addition, within the field of nanoparticle arrangement, direct current (DC) spray coating strategies and radio frequency (RF) spray coating strategies are well-known strategies for planning silver nanoparticles and gold nanoparticles with cellulose fabric to create promising nanocomposites [59].

## 3. Silver Ions

The preparation of silver nanoparticles on different surfaces assists the presence of unique properties that are absent in bulk or in micrometric range. Silver nanoparticles were prepared through various methods, such as electrochemical, photochemical, and chemical reduction, with the aim of a high yield and monodispersing within desirable ranges between 10 and 100 nm [60]. The existence of silver nanoparticles on the surface of polymeric materials can induce important modifications on their electronic, optical, and chemical properties [61]. Recently, noble metals were recognized as desirable antibacterial additives. In particular, special interest has been focused on Ag due to its strong antimicrobial activity against bacteria, fungi, protozoa, and viruses. Among the silver nanoparticles-based composites, tremendous efforts have been devoted to constructing antibacterial cellulose/Ag nanocomposites for various applications. Silver, either as nanoparticles (Ag^0^), oxides (mainly Ag_2_O), or in ionic forms (Ag^+^), has a high antimicrobial activity against bacteria, fungi, and viruses [62]. Recently, silver nanoparticles were applied in various medical devices and as a disinfectant against hospital wastewater [63]. Silver nanoparticle (AgNP)-doped cellulosic materials display strong antimicrobial activity against bacteria, fungi, protozoa, and viruses [64].

The antibacterial capacity of Ag NPs is affected by their size and stability in practical applications. For this reason, appropriate Ag NPs construction technologies, such as silver nanoparticles, are recognized to coagulate and aggregate in solution, which leads to changes in their size and morphology, and, consequently, their antibacterial properties may reduce. Many trials were carried out to develop an effective technology to anchor silver nanoparticles to a supporting polymer to avoid their aggregation and, consequently, sustain their activity [65].

The immobilization and encapsulation of silver nanoparticles with polymer matrices, including polymer particles [66], micelles [67], and hydrogels [68], have been described as promising techniques to avoid aggregation. Moreover, silver nanoparticles could be encapsulated in different natural polymers to preserve their activity [69]. The antimicrobial properties of the most described polysaccharides/silver composites were verified for a few bacteria. However, their biocompatibility has not been examined, and the absence of real studies about biocompatibility limits the applications of such composites. Therefore, the construction of new techniques to anchor silver nanoparticles to biopolymers, especially cellulose, is essential to evaluate the antimicrobial features and biocompatibility of these composites. Silver nanoparticles can be produced by physical methods, such as evaporation and condensation techniques and mechanical ball milling [70,71]. These methods form silver nanoparticles with impurities without uniform particle distribution. The chemical methods require the use of a capping agent, reducing agent, and reaction solvent, which are mostly non-sustainable chemicals and/or cumbersome operation procedures. The preparation of nanoparticles using polysaccharides as reduction and end-capping chemicals is another practical technique to decrease the environmental impact of using hazardous materials [72].

## 4. Preparation Methods for Cellulose/Silver Nanocomposites

There are various techniques for producing silver nanoparticles, each of which produces particles with different properties, such as size distribution, stability, shape, diameter, or contamination levels. Silver nanoparticles can be made in different solutions, including polysaccharides, though aqueous solutions are the most reported. The most common way to create cellulose–silver nanoparticles is to reduce silver nitrate with ascorbic acid; other frequent reduction agents include sodium borohydride, ethanol, sodium citrate, and ethylene glycol. Silver ion reduction is also occurred by UV light or polysaccharides, such as cellulose [73,74,75].

### 4.1. Using Mutual Solvents or One Post-Synthesis

Ionic liquids were used as solvents for both silver ions and biopolymer. Cellulose and keratin were dissolved in 1-butylmethylimmidazolium chloride and silver was added after complete dissolution. The formed silver can be continued as ionic silver (Ag^+^) or fully reduced to metallic silver (Ag^0^) by a suitable reducing agent. The ionic liquid assisted the homogenous distribution of the two polymers in the formed composite homogenously. Tran et al. investigated the antibacterial activity and biocompatibility of the cellulose/keratin blends containing either Ag^+^ or Ag^0^. The silver concentration is the main parameter in the biocompatibility and antibacterial measurements. At the same silver content, the blends containing Ag+ are relatively more toxic for bacterial and human cells than the blends containing Ag^0^ nanoparticles [65].

Y Fujii et al. described the preparation of porous cellulose through dissolving in 1-butyl-3-methylimidazolium chloride and N, N’dimethylformamide. The dissolved cellulose was dropped into 1-butanol to obtain particles with high specific area. These porous particles were functionalized via oxidation by TEMPO, which generates new carboxylic groups. Composite cellulose/Ag particles were prepared by ion exchange of carboxylate groups to Ag cations, followed by the reduction reaction. To eliminate the unbound cellulose/Ag particles, the obtained cellulose/Ag particles were extensively washed with water. Immobilization of as-prepared AgNPs on cellulose particles occurred by covalent bonds for improving recyclable catalytic performance, which was estimated by observing the reduction of 4-nitrophenol, as shown in Figure S7 [76]. Polyethylenimine acts as a stabilizing agent in the silver reduction reaction and performs three functions: changing the pH of the solution for accelerating silver ion reduction to metallic nanoparticles, preventing the agglomeration of silver nanoparticle, and preventing cellulose/*N*-methylmorpholine-*N*-oxide (NMMO) degradation during the synthesis [77].

The antibacterial BC/silver nanocomposites were prepared by one-step facile environmentally friendly green approach in the presence of green tea as reducing agent (Figure S8) [78]. Shaheen & Fouda demonstrated the preparation of Ag nanorods in the presence of alkali by CNCs without any surface modification (Figure S9). CNCs act as a reducing and stabilizing agent for the assembly of Ag nanorods [79]. To deposit AgNPs on CNCs, Hanif et al. developed an environmentally friendly one-pot approach that uses a biocompatible polyvinylpyrolidone (PVP) as a reducing agent (Figure S10). The new approach demonstrated homogeneous in situ AgNP deposition on CNCs. The hydroxyl (OH) group of CNCs and the amide group of PVP have a high possibility to form a hydrogen bond. The coordination bonds between PVP and silver ions, as well as silver ion reduction, began to generate silver nanoparticles during stirring. The produced CNC/PVP/Ag nanohybrid exhibited high colloidal stability (8 months), anti-biofouling, and antibacterial properties. The hydroxyl groups on the CNCs’ surface enables in situ reduction of silver salt for controlled and uniform deposition of AgNPs on individual CNCs and decoration of PVP at room conditions in the one-reaction vessel [80]. A uniform cellulose/Ag nanocomposite film was effectively produced with *N*,*N*-dimethylacetamide (DMAc) as a reducing agent in the presence of PVP-K30. CANF and mercaptopropyltrimethoxysilane (MPTS) were cast in the same container with LiCl to generate the AgNP/PVP/DMAc solution, Figure S11. Because of the AgNPs, the CANF have improved tensile strength, thermal stability, and antibacterial properties. Therefore, the silver-loaded cellulose films are suitable for applications in bacterial barrier and food packaging [81].

### 4.2. In Situ Reduction

The in situ production of silver nanoparticles in a cellulose fiber starts with the adsorption of the silver salt onto cellulose, followed by a reduction to metallic silver. It has been reported that this technique is the most common preparation technique for the construction of silver/cellulose composites. The hydrothermal in situ reduction method is a viable alternative for incorporating silver nanoparticles into cellulose solutions, which were then dry-jet wet-spun to produce cellulose textiles [82]. For example, Jatoi et al. investigated the fabrication of AgNPs on cellulose nanofiber using thermal treatment and DMF as a reducing agent for antibacterial activity [83]. The aggregation of nanoparticles is reduced by the immobilization technique. Stability of metallic nanoparticles was accomplished through the steric hindrance by attaching big molecules (capping agents) to the surface of the nanoparticles, such as polyvinyl alcohol (PVA) [84] and polyvinylpyrrolidone (PVP) [85]. Moreover, gelatin biopolymer has been employed as capping agent for AgNPs [86]. Spagnol et al. successfully incorporated cellulose nano-whiskers functionalized with carboxylate group into PVA polymer matrices and poly (Nisopropylacrylamide), as shown in Figure S5. All films with silver nanoparticles showed antimicrobial activities against gram-positive and gram-negative bacteria [87].

**Figure 4:**
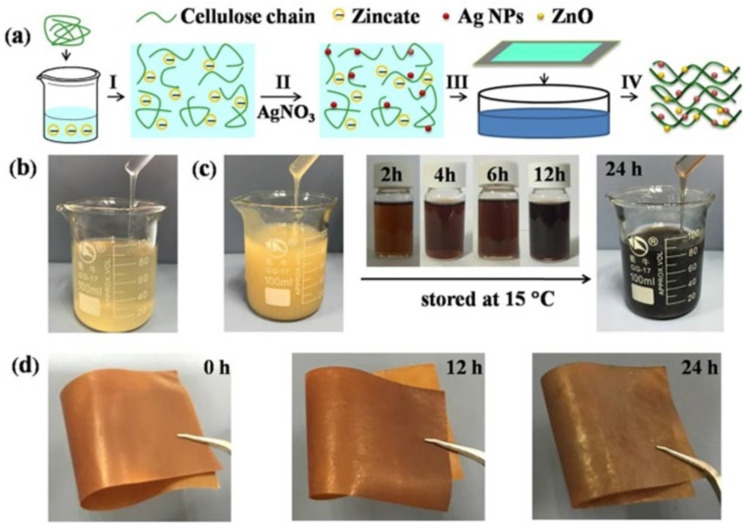
(**a**) Preparation of the Ag/ZnO decorated cellulose nanocomposite films: (I) cellulose dissolving in aqueous NaOH/urea/zincate solution, (II) Ag^+^ reduction in cellulose dope, (III) casting and coagulation of films, (IV) the produced nanocomposite film. (**b**) Photographs of dissolved cellulose dope, (**c**) after different storage time, mixing of AgNO_3_−cellulose dope system, and (**d**) resultant nanocomposite films [88]. Copyright © 2018, American Chemical Society.

Cellulose nanocomposite films were prepared by mixing a cellulose NaOH/urea/zinacate with AgNO_3_ (Figure 4). The mixture gelation is delayed by the presence of zincate, making the reduction of silver ions in a cellulose dope feasible, with good dispersion and mean diameter of 16.5 nm. The produced nanocomposite has tensile strength, elongating at break and Young’s modulus of 55.2 MPa, 4.7%, and 3.6 GPa, respectively. Furthermore, at lower concentrations, the Ag/ZnO nanoparticles decorated cellulose nanocomposite showed good cell compatibility, which was sufficient to remove both *E. coli* and *S. aureus* bacteria within 3 h [88].

**Figure 5:**
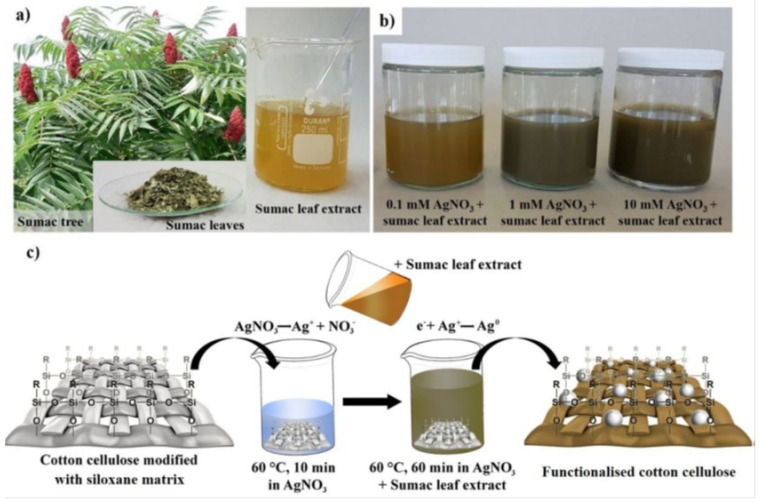
(**a**) preparation of sumac leaf extract; (**b**) different concentrations of AgNO_3_ with sumac leaf extract colloidal solutions; (**c**) schematic representation of in situ biosynthesis of AgNPs on cotton cellulose [89]. Creative Commons CC-BY license, 2021.

A novel, simple, and eco-friendly procedure for fabricating multifunctional antibacterial and ultraviolet (UV) protective cotton surfaces was developed using cellulose fibers with a polysiloxane matrix, followed by green in situ biosynthesis of silver nanoparticles (AgNPs) in the presence of sumac leaf extract as a reducing and stabilizing agent. It was carried out in a two-step procedure. In the first step, the cotton samples treated with the siloxane matrix were immersed in AgNO_3_ solution. During the second step, the sumac extract solution was added to the samples (Figure 5); the combination between AgNPs and aromatic phenolic compounds of sumac leaf extract provided excellent UV protection and antimicrobial activity [89].

Chemicals have a significant role in waste management issues. As a result, bioreduction is the safest and most practical solution available. Recent studies have shown in situ synthesis of silver nanoparticles in a cellulose matrix employing Marrti and Neelagiri leaf extract, Tecoma stans extract, soy leaf, and curry leaf (Murrayakoenigii) extracts (Figure S6). Despite being environmentally safe, all of the previous mentioned processes require a long time to make nanoparticles, ranging from 1 to 6 h.

Other studies have investigated the antimicrobial efficiency of cotton fibers loaded with silver nanoparticles (AgNPs) by using natural extracts of Eucalyptus citriodora and Ficus bengalensis, exhibiting superior antibacterial activity even after several washings, thus suggesting a valid use as medical textiles for infection prevention [90]. Moreover, the fabrication of silver and copper nanoparticles in cellulose nanofibers was investigated in two ways: chemical reduction and UV excitation. Silver and copper nanoparticles loaded on cellulosic scaffolds displayed good antibacterial capabilities against *B. subtilis* and *E. coli* [91].

Alahmadi et al., used low cost, water-soluble additives, such as carboxymethyl cellulose, α-cellulose, and aminocellulose, in a small amount with in situ chemical reduction by NaBH_4_ for the preparation of silver/cellulose nanocomposites to inhibit the aggregation of silver nanoparticles and significantly increase their concentration on the surface of the cellulosic fibers of the paper sample [92]. Liu et al., demonstrated that Ag nanocluster synthesized on BC can be obtained through a two-step process. The BC hydrogel having a 3D network-like structure (as the backbone material) was first immersed in an AgNO_3_ solution, as the Ag precursor, allowing Ag^+^ ions to be adsorbed on the surface of the nanofibers by interacting with the –OH groups on the BC surface [93]. The second step was the introducing of NaBH_4_ and dihydrolipoic acid (DHLA) as a reducing agent and protecting molecules to the reaction system, respectively, in which the as-formed DHLA–Ag^+^ complex could be reduced by NaBH4 via the S–Ag^+^ interaction, resulting in the in situ production of DHLA-protected Ag NCs [93].

### 4.3. Electrospinning and Electrospraying

Electrospinning is a practical technique for creating fibers out of polymer solutions with specific diameters. The method depends on the stretching of a droplet having the polymer at a definite applied voltage. A charged solution jet is shaped and extended by electrostatic repulsive forces to form a regular fiber [16]. The incorporation of [Bmim]BF4 and silver nanoparticles to an ethylcellulose matrix generated by electrospinning were studied for using in biomedical applications to measure oxygen levels. The nanofibers provided several benefits, including improved sensor dynamics, increased surface area, and tailored sensitivity. Furthermore, the materials exhibited long-term stability over a year [94]. Zheng et al. demonstrated [Emim]Ac as solvent to dissolve cotton, and in an electrospinning solution to produce nanocellulose fibers. The composites showed increased surface area and significant antibacterial activity [95]. Hasham et al. demonstrated the preparation of regenerated cellulose nanofibers from cellulose acetate containing different concentrations of the Hap, which are treated with AgNO_3_ solutions using in situ methodology approach (Figure S6) [96].

Cellulose acetate (CA) microfibers were prepared by electrospinning, then converted to regenerated cellulose (RC) by immersing for 24 h in 0.05 M NaOH alcohol solution. The RC microfibers were treated by 5% NaIO_4_ aqueous solution for oxidation to dialdehyde cellulose. The oxidated (ORC) microfibers were immersed in 0.5 M AgNO_3_ at room temperature for 2 h, then gently washed with water to remove the excess of AgNO_3_. Finally, the resultant color of wet microfibers turned to brown at 60 °C [95]. Srivastava et al. aimed to evaluate the anti-biofilm and antimicrobial properties of an electrospun cellulose acetate nanofiber mat containing green synthesized silver nanoparticles (CA-g-AgNP nanomat) by the physical adsorption method [97].

The addition of g-AgNPs into the CA nanomat improves the antibacterial activity, resulting in greater than 50% reduction in biofilm formation. The CA-g-AgNP nanomat also has 80% cytocompatibility. This green-synthesized CA-g-AgNP nanomat would help to reduce the financial burden of healthcare associated with biofilm formation and infections. San Keskin et al. employed the electrospinning method for the preparation of cellulose acetate nanofibers (CA-Nf) loaded with biogenic silver nanoparticles using *Lysinibacillus* sp. NOSK cell-free extract as a reducing agent for the silver nanoparticle production [98]. The aqueous CA solution was dissolved in DCM/MetOH (4/1, *v*/*v*) mixed solution to prepare neat non-porous CA nanofibers. The nanoparticles were dispersed in a previous solution by sonication. Then, suspension stirred overnight before being placed into syringes and began electrospinning process. Copper was efficiently protected by electrospun CA nanofibers/AgNPs in both abiotic and biotic marine environments. A novel electrospun fiber mat composition [CA/PEG/AgNPs] with improved functional and antibacterial properties was created by reducing AgNPs in situ via a chemical reaction with the electrospinning solvent 2:1 acetone/DMAc and stabilizing them with PEG to prevent particle agglomeration. The fiber mat containing PEG showed increased swellability higher than without PEG [98]. Wang et al. used the electrospinning method to incorporate CMC/PEO nanofibers with AgNPs, and no other reducing agent was needed. The produced AgNPs have a spherical shape and diameter of 5 nm. CMC/PEO/AgNO_3_ blend can be applied as wound dressings or anti-adhesion membranes [99].

Using bacterial cellulose nanocrystals (BCNs) as a model substrate was investigated by Musino et al. They investigated the interactions between the cellulose surface and Ag nanoparticles (AgNPs), where the Ag^+^ ions are reduced chemically by NaBH_4_ in ice to minimize its decomposition and homogeneous hybrid suspensions of BCN/AgNP are generated regardless of whether the BCNs are quasi-neutral, positively charged (ABCNs), or negatively charged (TBCNs). The characterization of BCN/AgNP hybrids identified the –OH surface groups as nucleation points for AgNPs, confirming that the surface charges only improve the accessibility to OH groups [100]. The produced nanohybrid can be employed in many applications, such as food packaging, paints, sensors, biological imaging, and surface treatment.

**Figure 6:**
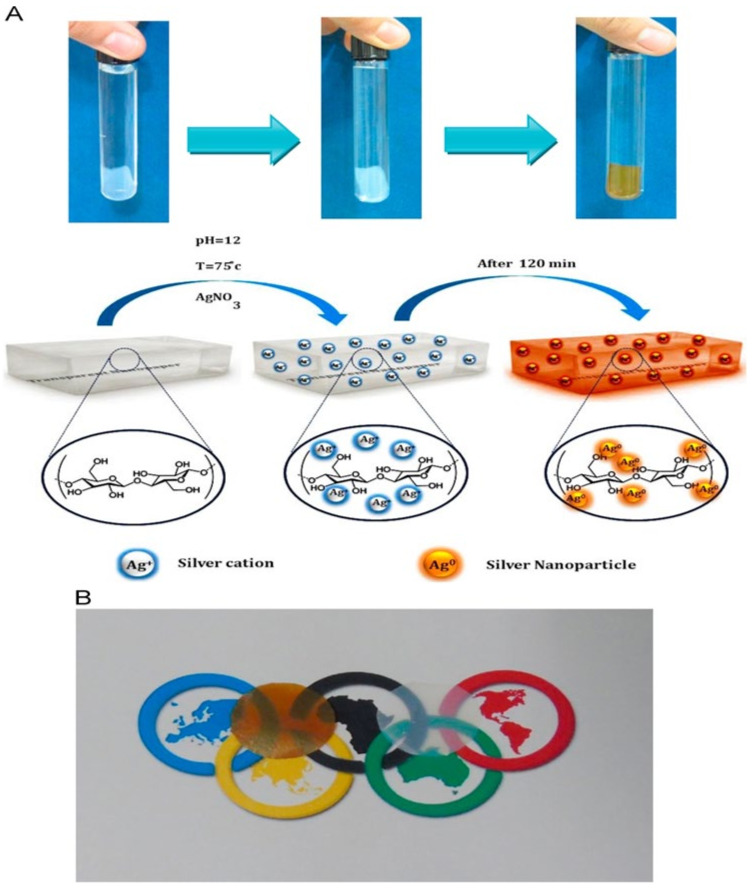
(**A**) Schematic representation of the fabrication of ESNPs and (**B**) a transparency demonstration of dried films of ESNP on top of the Olympic symbol (left) and bare nano-paper on top of the Olympic symbol (right) [101].

Nanoparticles were directly synthesized in situ by the reduction of adsorbed silver ions by hydroxyl groups within transparent bacterial cellulose films without adding any external linking, stabilizing, or reducing agents. A new bio-nanocomposite, i.e., embedded silver nanoparticle into a transparent nano-paper (ESNPs), was created thanks to this process as a way of fabricating the ESNPs with high performance as optical sensors. The effect of pH of solution, AgNO_3_ concentration, AgNO_3_/nanopaper mass ratio, temperature, and reaction duration on the optical properties of ESNP were studied by Nahid et al., 2015 [101]. Figure 6A shows a schematic representation of the fabrication process used in this study, while Figure 6B demonstrates the transparency of dried films and ESNP films. The presence of AgNPs in transparent ESNPs resulted in the amber color of this figure.

In the future, silver could be assembled in the form of micro and/or nanoparticles to electrospun fibers to design micro and/or nanostructured platforms with a higher control of antibacterial functionalities without altering the structural properties of the fiber network [102]. Currently, manufacturing technologies, such as electrospraying, are currently used for the fabrication of cellulose-based nanoparticles for the encapsulation of a large variety of active agents, including metal ions [103]. In particular, the morphological signals typically exerted by nanofibers can be complemented by the non-specific capability of nanoparticles to fight microbial resistance [104]. In perspective, the application of electrospraying as additive technique may be suitable to transform electrospun fibers into a more efficient delivery system [105], overcoming some limitations on the use of silver, mainly due to the level of toxicity for human health and biological environment.

### 4.4. Additive Manufacturing

Electric assisted additive manufacturing (EAM) includes a selected group in the wide collection of process methodologies based on a layer-by-layer deposition of polymers by the support of automated machines or computer aided design to create three-dimensional substrates [106]. In few years, these technologies have been efficiently adapted to the use of natural polymers with peculiar biomechanical properties, such as cellulose and its derivatives, for the formulation of innovative composite hydrogels with smart bio-functionalities (i.e., structural reinforcement [107], stimuli responsivity [108], antibacterial properties) for different biomedical uses (i.e., wound dressing [109], prostheses [110], and tissue engineering) [111].

In the past, AM technologies have been variously used to process cellulose-based composites with desired structural resolution by adapting the basic principles of other conventional techniques (i.e., extrusion, powder sintering, photo-polymerization, and sheet lamination methods). For instance, conventional extrusion was optimized to mix short cellulose fibers with other polymers and/or other agents, including metal ions (i.e., silver) or bioactive particles (i.e., calcium phosphates, bioglasses), to form a multicomponent filament with improved functional properties and biocompatibility [112]. In order to overcome some limitations ascribable to struct resolution and surface defects, melt electrospinning writing based on the combination of electrospinning with larger scale melting extrusion is recently emerging as the most interesting EAM technology to realize highly ordered 3D architectures. From Dalton’s pioneering studies on the interaction of melt polymers with electrostatic forces [113], this technique may be successfully used to write synthetic and natural polymers by the use of automated machines able to mechanically control spinneret translation along x-y axis in order to realize fiber dispensing systems in the form of 3D scaffolds with controllable architectures and patterns [114]. More recently. Xu et al. utilized the melt electrospinning process supported by laser heating to spin cellulose/BmimCl solution homogeneously mixed with either water or ethanol [115]. Flexible gel rods based on cellulose were prepared via crystallization process or film casting, A laser-based heating system (power 10 W/cm^2^) was used in combination with voltage to collect electrospun fibers onto a nitrogen cooled metallic plate (−40 °C). Fine fibers of 1 µm generated from a high degree of polymerization cellulose allowed forming highly reproducible architectures. In perspective, this solvent free technology could be really advantageous for the entrapment of silver nanoparticles, limiting the chemical interaction at the interface, typically due to the use of chemically aggressive solvent, that can alter the native oxidation layer of the surface nanoparticles and, consequently, their antibacterial properties [116].

#### 4.4.1. Electro Aided Dropping (EAD)

**Figure 7:**
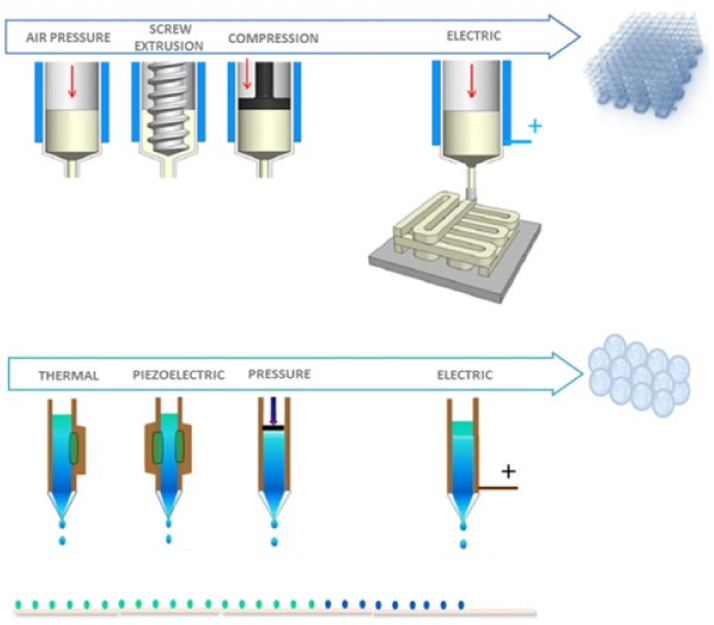
Scheme of different additive manufacturing technologies used for the processing of nanocellulose-based materials—image adapted from [119].

More recently, alternative methodologies inspired by AM technologies are emerging to print cellulose derivates and composites by liquid dropping of gels/inks driven by the application of electrical forces [117]. This approach enables the fabrication of bio-hydrogels with different size scales to design smart platforms able to better mimic the behavior of biological structures and control their response under environmental stimuli [118]. By the ejection of liquid inks through a micrometric nozzle (Figure 7), these processes allow forming spatially organized patterns by the guided deposition of thin droplet layers (0.2 to 2 µm) with high resolution (up to 40 µm as line width). An accurate control of ink properties (viscosity, surface tension specific range, colloidal stability) is crucial to allow a correct droplet ejection and prevent undesired nozzles clogging. Meanwhile, the use of electrical/thermal conductive elements, such as metallic nanoparticles homogeneously dispersed into the ink, is especially promising to support the electro-aided dropping (EAD) mechanism for the fabrication of innovative smart platforms for bioelectronics and nanomedicine.

The initial studies were focused on the EAD of cellulose nanocrystals dispersion obtained from the acid hydrolysis of the cellulosic fibers for the deposition of rigid rod-like particles with a length ranging from 100 to 500 nm and a diameter ranging between 5 and 30 nm to form self-standing films [120]. More recently, these techniques were adapted to produce hybrid systems loaded with metallic particles (i.e., silver) with antibacterial or electro-conductive properties [121]. In this context, the studies referred especially to the additive dropping of conductive phases directly onto nano-cellulose substrates [122,123]. To date, only few works proposed the direct dropping of cellulose-based conductive inks. For example, Koga et al. first emphasized the dispersing properties of nanocellulose to provide a conductive inkjet printable suspension of cellulose nanofibrils/carbon nanotubes [124]. More recently, Hoeng et al. suggested a new hybrid ink based on the combination of silver nanoparticles and cellulose nano crystals (CNC) as conductive slurry to fabricate nanostructured patterns for sustainable electronics that are characterized by higher electrical conductivity—about an order of magnitude more than conventional inks [125]. A relevant limitation in the use of EAD technologies concerns the ink viscosity: up to 10 centipoise excessive force may be required to eject drops [126], with clogging phenomena at the tip of small size nozzles. In this way, the recent customization of electrohydrodynamic processes may offer new opportunities to use polymeric solutions or slurries in a wider range of viscosities [127]. As a function of the solution properties, it is possible to control the drop formation assisted by the electrical forces, not only to generate continuous patterns but also to confine cellulose materials in micro or nanosized units with highly customizable surface area [128,129] suitable for the fabrication of innovative Ag release systems.

#### 4.4.2. 3D Ink Jet Printing

Cellulose and its derivatives are proven to be useful in the formulation of hydrogels and polymer-based composite materials (using both thermoplastics and thermosets) fabricated via 3D printing, having gained great attention in recent years. The process of 3D printing involves creating products from raw materials directly from a 3D digital model by layering the materials. By incorporating nanomaterials into polymer matrices using 3D printing, nanocomposites can be manufactured with improved properties, customized geometry, reduced design iterations time, and increased part integration, and can be done in two different ways. Adding nanomaterials to matrix material can be done automatically or manually, with stoppages for batch printing. Alternatively, the nanomaterial can be added to the host matrix first, and then the nanocomposite can be printed from the mixture [130].

**Figure 8:**
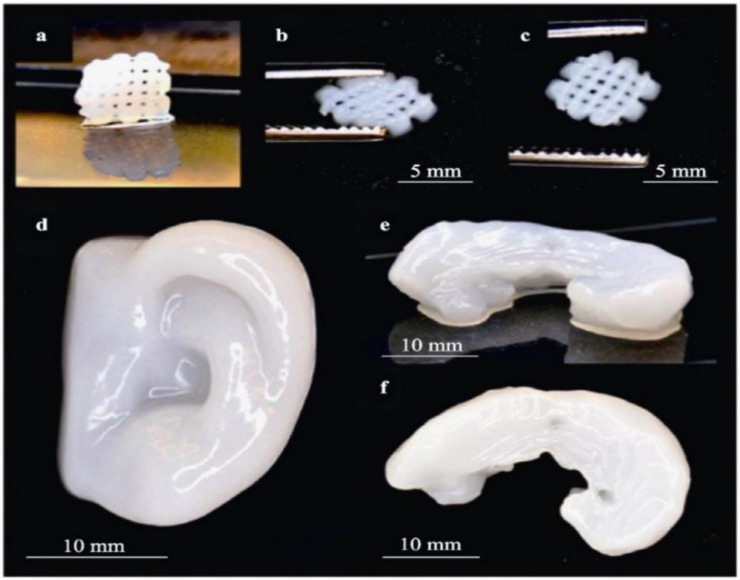
(**a**) 3D printed small grids (7.2 mm × 7.2 mm) with Ink 8020 after cross-linking; (**b**) shape of grid deforms while squeezing; and (**c**) it is restored after squeezing. (**d**) 3D printed human ear and (**e**,**f**) sheep meniscus with Ink 8020. Side view (**e**) and top view (**f**) of meniscus [137].

CNFs and CNCs are both commonly used as ink materials in direct 3D-printing-based technologies. A variety of conductive cellulose inks have been demonstrated as conductive inks for 3D printed electronic devices, including inks containing conductive fillers, such as graphene and carbon nanotubes. The conductive ink matrix is formed from cellulose, both as a substrate and a matrix. The biocompatibility of cellulose inks makes them an attractive candidate for biomedical applications. Inks formulated from cellulose have been developed recently to handle volume shrinkage due to the drying and evaporation of the dispersing solvent [131,132,133,134]. Other studies involve the implementation of different 3D printing processing methodologies that enable the fabrication of custom-made devices for several applications in repair and tissue regeneration [135,136,137] (Figure 8). In this view, nanocelluloses were recently recognized as an excellent 3D printing material for their ability to mimic the fibril network of collagen [138]. However, the current literature on the 3D ink jet printing of nanocelluloses (i.e., T-CNF) is currently scarce. Contrariwise, several efforts are promoting the combination of cellulose with other polymers (e.g., alginates) as a successful strategy to stabilize the 3D printed structure. [139]. For instance, alginates can be properly cross-linked with Ca^2+^, giving the opportunity to design 3D bioprinted scaffolds suitable for chondrocyte cells in cartilage tissue engineering [140].

Alternatively, a new formulation of silver nanoparticles/alginate/nanocrystalline cellulose was used in 3D printed scaffolds with large surface areas, improved mechanical resistance, and sustained antimicrobial and cytotoxic effects. The scaffolds were characterized on the basis of mechanical resistance, water content, morphological characteristics, and silver distribution. Based on the in vitro test results, the scaffold showed comparable antimicrobial potency against *S. aureus* and *P. aeruginosa* as a function of the AgNP concentration (minimum inhibitory moncentration of 10 mg/mL) [141].

## 5. Properties and Applications of Cellulose/Silver Composites

The preparation, properties, and applications of different cellulose/silver composites are summarized in Table 1.

### 5.1. Drug Delivery

Oral delivery is reported as an efficient strategy for drug administration. However, this strategy faces many challenges, such as low stability in the gastrointestinal tract and poor permeability through the intestinal epithelium, which minimize the bioavailability of therapeutic molecules. Many efforts have been employed for engineering efficient oral drug delivery systems based on natural polymers that significantly enhance oral bioavailability. Functionalized carboxymethyl cellulose and silver nanoparticles nanocomposite material has been prepared by an in situ deposition method for the control of transdermal drug delivery. Cross-linked CMC-based hydrogel was synthesized by free radical polymerization via the grafting of [2-(methacryloyloxy) ethyl] trimethylammonium chloride] on CMC in the presence of a diethylene glycol dimethacrylate (DEGDMA) cross-linker for preparing a porous and rigid hydrogel with high efficiency for drug loading and release. The cross-linked CMC-based hydrogel exhibited different binding sites, which can form a physical/covalent bond with the drug molecules. Moreover, the drug molecules can be attached via hydrogen bonding or physical interactions. It has been reported that the presence of silver nanoparticles with chemically cross-linked CMC would promote the drug loading efficiency and controlled release properties. Moreover, silver nanoparticles have a low probability of agglomeration during synthesis and a higher accessible area for binding the drug molecules.

B. Mandal et al. prepared hydrogel comprised of in situ formed silver nanowires-deposited chemically cross-linked carboxymethyl cellulose as a promising anticancer drug-curcumin carrier. The nanocomposite has the ability to encapsulate both hydrophobic/hydrophilic transdermal drugs. The in vitro release of curcumin proposes that the nanocomposites improved the penetration power of the nanocomposite and released the drug in a sustained way (~62% for curcumin released in 4 days) [151].

### 5.2. Wound Healing

Wound dressing is a vital biomedical material for wound protection and healing promotion. The ideal wound dressing displays intrinsic biomedical properties, such as biocompatibility, providing a moist environment, absorbing exudates, and great mechanical properties [53,152]. In the wound healing applications, silver ions must be retained inside a solid support to apply over the affected area. Bacterial cellulose is more favorable for wound healing applications due to its high porosity and water permeability [153]. Sudipto Pal et al. prepared bacterial cellulose decorated with silver nanoparticles through the facile green synthesis of silver nanoparticles inside the porous three-dimensional bacterial cellulose by UV light irradiation. The authors proved the deposition and chemical bond formation of silver nanoparticles onto the BC gel network (Figure S12). An antibacterial study showed a high bacteria-killing performance with low silver release after a long soaking time, which confirms the stability of the Ag nanoparticles inside the composite matrix [20].

C. Wu et al. modified bacterial cellulose by 2,2,6,6-tetramethylpiperidine-1-oxyl radical (TEMPO)-mediated oxidation for preparing oxidized cellulose nanofibers, which was subsequently ion-exchanged in an AgNO_3_ solution. The silver nanoparticles have a diameter of ~16.5 nm and were prepared by in situ thermal reduction without using a reducing agent. The results showed that the silver nanoparticles continuously released with a rate of 12.2%/day at 37 °C in 3 days. Moreover, the prepared nanocomposite showed high biocompatibility (cell viability >95% after 48 h of incubation) and presented high antibacterial activities of 100% and 99.2% against *E. coli* and *S. aureus*, respectively [145].

### 5.3. Electric Conductivity

The preparation of cellulose/silver conductive sponges with predictable and controllable performances was reported. Cellulose nanofibrils and silver nanowires composites were prepared by the directional freeze-drying technique. The prepared sponge showed a high compressive stress of 24.5 kPa, low percolation threshold of 0.1 vol % silver nanowires, and high electrical conductivity of 1.52 S/cm. These unique features are generated from the presence of well-aligned and oriented channels, and dense and random pores. This sponge composite material is expected to extend to various applications, such as electronic devices, for a smart switch or EMI shielding [144].

Tae-Won Lee et. al. prepared silver nanowire covered cellulose papers with a hierarchical morphology through a dip-coating technique. The results exhibited that Ag nanowires are coated dominantly on the paper surfaces and partially in the inner parts of the fibers. The electrical conductivity of the cellulose/silver nanowires papers in the in-plane direction increases significantly from 0.34 S/cm to 67.51 S/cm by increasing the dip-coating cycle from 1 to 50, respectively. Moreover, the silver nanowires/cellulose paper with an apparent electrical conductivity of 67.51 S/cm displays a high EMI SE of ~48.6 dB at 1 GHz [148].

P. Boberof synthesized new free-standing multifunctional composites via in situ one-step chemical polymerization containing cellulose nanofibers covered with polypyrrole and silver nanoparticles as electroconductive material with antimicrobial properties. The author showed that the incorporation of cellulose nanofibers into polypyrrole developed the performance of the formed films with good mechanical and electrical properties. Moreover, the composite has an inhibition effect against the growth of gram-positive bacteria. The antimicrobial activity presents the silver composites in various applications aimed at biomedical treatments and diagnostics [154].

Sukun Zhou prepared high porosity and low density microfibrillated cellulose aerogels through freeze-drying, which were used as templates for the preparing of micro fibrillated polypyrrole/silver nanoparticles through a simple dip-coating method. The obtained aerogels showed improved antimicrobial and electrical conductive properties due to the combination of polypyrrole and silver. These properties make the hybrid aerogels promising candidates for wound healing, energy storage, and pressure sensing applications [155].

### 5.4. Thermal Conductivity

Thermal management materials (TMMs) with high thermal conductivity (TC) have recently emerged in the electronic field. They can eliminate the redundant heat produced by high-power integrated circuits in the electronic devices [156]. Polysaccharides were reported as promising matrices for thermal management materials due to their ease of processing, light weight, and low cost. However, the low TCs of these polymers cannot meet the demands for fast heat conduction. These polymers were blended with silver nanoparticles to enhance the thermal conductivity [157]. For example, Z. Shen and J. Feng prepared a flexible nanofibrillated cellulose/silver composite film with high thermal conductivity through an in situ coating process. The Ag nanoparticles were homogeneously coated on the surface of nanofibers for producing films with high strength and excellent flexibility. The composite films containing only a 2.0 vol% of Ag displayed a high in-plane thermal conductivity value of 6.0 W/(m·K). The highly thermally conductive composite can be applied as lateral heat spreaders in flexible electronic equipment [158].

### 5.5. High-Performance Textiles

Modern textile preparation needs different functionality in order to be reasonable on the market. Metal nanoparticles, such as silver nanoparticles, showed a distinction for the functionalization of modern textiles. Silver and gold nanoparticles have received considerable interest as alternatives to textile dyes and improving the antimicrobial properties of the natural fibers [159,160]. The utilization of silver nanoparticles is due to the high surface area and a phenomenon referred to as localized surface plasmon sonance [161]. Metal nanoparticles interact with the electromagnetic field of the light, which activates a collective oscillation of the nanoparticles’ surface electrons. The absorption of light makes the nanoparticles show various colors, which depend on the shape and the size of the nanoparticles [162]. Recently, in situ reduction methods were employed using ultraviolet light or hydrothermal conditions in the presence of plant derivatives as reducing agents to synthesize noble metal nanoparticles [163]. The reducing end groups of cellulose can react with metal ions for producing nanoparticles that could be stabilized in the fiber matrix as spherical nanoparticles. The prepared nanoparticles by this method are more stable, and, consequently, they are appropriate for textile applications.

Simone Haslinger et al. prepared cellulose-based textiles through the incorporation of Ag nanoparticles into dry-jet wet-spun man-made cellulose fibers. Bleached birch prehydrolyzed kraft pulp worked as a reducing agent for silver nitrate (AgNO_3_) for producing metal nanoparticles via a green in situ reduction method. The pulp was dissolved in ionic liquid and spun to staple fibers. It is clear that the tensile properties of the regenerated fibers were not affected by the presence of the nanoparticles. The incorporating of silver nanoparticles in the cellulose reduced their release into the environment, thus providing a sustainable technique for textile production [74].

### 5.6. Photocatalytic Properties

The fabrication of porous photocatalysts via a sustainable and simple technique is still a challenge. The facile methods for the preparation photocatalysts with a promising morphology and porous construction are highly required. The fabrication of metal-semiconductor oxide, such as TiO_2_/Ag, is a promising research area to develop the photocatalytic performances of semiconductor oxide photocatalysts. The electron transfer from semiconductor oxides to metal nanoparticles was suggested to decrease the number of electron/hole recombinations and, consequently, can develop the photocatalytic activities of semiconductor oxide-based catalysts. Moreover, the porosity can increase the diffusion of pollutants during the channels, and the metal particles act as electron sinks. It has been reported that the photocatalytic performance of semiconductor oxides will be attained by the controlled porosity and addition of metal nanoparticles [143]. Y. Dong-Hui established an effective method for the preparation of TiO_2_/Ag nano-sponge composite using template-cellulose fibers associated with the surface sol−gel process. The Ag nanoparticles are deposited on the TiO_2_ nano-sponges via the UV irradiation photoreduction of silver nitrate solutions. The photodegradation of organic dyes, RhB, and salicylic acid by TiO_2_/Ag porous material was attributed to the high dispersion and small nanoparticle size of Ag nanoparticles, as well as the strong interaction between the metallic Ag particles and TiO_2_ samples [143].

### 5.7. Sensors

Scientists have developed sensitive and suitable techniques for the measuring of vital materials in environmental, pharmaceutical, and biological fluids, such as high-performance liquid chromatography, mass spectrometry, and fluorescence examination [147]. These techniques are usually accompanied with conditions such as high temperatures, specified instruments, and using toxic reagents to improve the detection of trace components. Recently, colorimetric sensing based on silver nanoparticles has been presented as a favorable method for sensing reagents because of their high extinction coefficient and range-dependent optical properties [82]. Z. Yu. et. al. prepared silver nanoparticles by using TEMPO-oxidized cellulose nanofibrils as a reducing agent and stabilizer under slight aqueous conditions for producing stable CNF/silver nanoparticles, which is applied for the rapid and selective detection of L-cysteine. Silver nanoparticles with a diameter range of 8−25 nm were formed with cellulose nanofibers, which is efficient for the colorimetric detection of thiol holding cysteine. The efficacy of the method depends on the surface plasmon resonance of the silver nanoparticles and the aggregation effect of silver nanoparticles. Cysteine interacted with silver nanoparticles for transferring the yellow color of the CNF/silver nanoparticles to purple. The prepared suspension presented high sensitivity and the rapid and selective detection of cysteine among 20 amino acids, as shown in Figure S13 [147].

**Figure 9:**
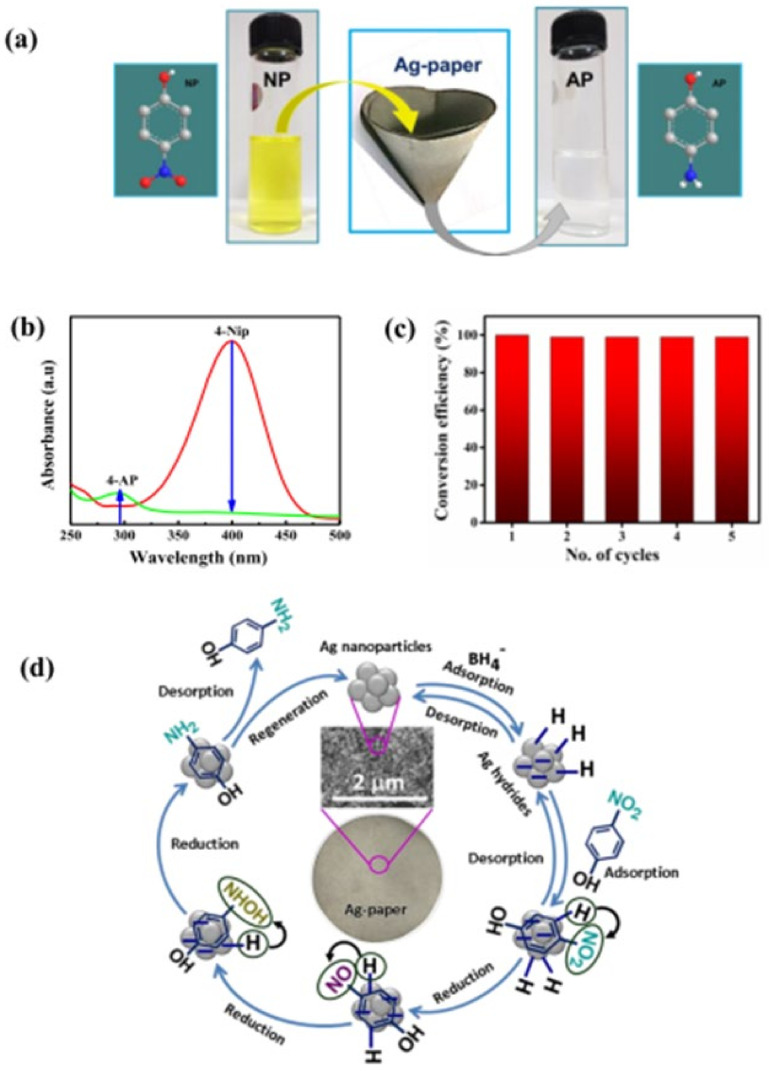
(**a**) schematic illustration of 4-NP reduction using Ag paper catalyst, (**b**) UV-vis absorption spectra of 4-NP before and after the Ag paper, (**c**) conversion efficiency for five repetitive cycles, and (**d**) schematic of a possible reaction mechanism.

D. Das suggested the deposition of silver nanoparticles onto filter paper for a controllable method. The filter paper/Ag nanocomposites were examined as an enhanced Raman scattering (SERS) and with detection limits down to pico- and nanomolar concentrations with an enhancement factor of 1.42 × 10^10^ and 0.695 × 10^6^ for rhodamine 6G and rhodamine B, respectively. The same SERS substrate was reused five times by simply rinsing it with water (Figure 9). Moreover, the filter paper/silver nanoparticles presented as an efficient and reusable catalyst for 4-nitrophenol reduction, and its duality as a SERS substrate should be emphasized [149].

### 5.8. Food Packaging

Biopolymers have been reported as promising materials for food packaging instead of traditional synthetic plastics. Among them, cellulose film is suggested to replace synthetic plastic-based film due to its good barrier property and the high mechanical property. However, antibacterial properties are required to improve the storage environment of food and lengthen the preservation time [164]. The most applied technique for improving the antibacterial properties of food packaging is the incorporation of an antibacterial agent or fillers into the applied film [165]. R. Gu et al. investigated the hyperbranched polyamide-amine as a template, reducing agent, and stabilizer to prepare silver nanoparticles in situ that anchored onto oxidized cellulose to create a regenerated cellulose film. The produced film showed a significant improvement in the mechanical, barrier, and antibacterial properties. The most important result is the ability of hyperbranched polyamide-amine to inhibit the release of silver nanoparticles from regenerated cellulose film and decrease the health threats [150]. Silver nanoparticles have been suggested as efficient antibacterial agents for the construction of antibacterial food packaging films [166]. Silver ions react with thiol groups on bacterial cell membrane proteins and consequently damage their ability to transport substances. As a result, silver nanoparticles inhibit the growth of a variety of pathogenic bacteria. It is important to immobilize the silver nanoparticles onto the films applied for food packaging to prevent their release and reduce their potential harm to the human body.

## 6. Conclusions

Cellulose is a versatile polymer for developing novel composites and devices for different application areas due to its large availability in nature, biodegradability, and biocompatibility. Recent discoveries on new formulations—i.e., nanofibrillar cellulose and bacterial celluloses—currently allow for the production of a large variety of cost-effective systems with higher sustainability and renewability. In this context, their combination with silver nanoparticles offers the unique opportunity to design a wide set of functional nanocomposites with tunable features (i.e., surface-to-volume ratio, mechanical stiffness, thermal expansion properties) by the implementation of tailored preparation methodologies. To date, the most common methods to prepare cellulose/silver nanocomposites consist of the in situ reduction of silver in the presence of cellulose. However, novel approaches based on green chemistry, or the use of ecofriendly procedures, are forcefully emerging to promote the use of green solvents for the synthesis of stable silver compounds, thus overcoming the main limitation of toxic chemicals on the final product. In this regard, recent studies have demonstrated that bacterial celluloses with high porosity and water permeability of nanofibrillar celluloses (i.e., CNF) can be variously combined with silver nanoparticles with recognized abilities to promote drug loading efficiency, controlled release properties, and antimicrobial properties for favorable use in wound healing and drug delivery applications. Meanwhile, emerging approaches based on the application of external electrical forces and additive manufacturing principles (i.e., electrospinning, electro fluid dynamic, 3D printing) can be easily adapted to in situ synthesis to promote a more homogeneous distribution of silver compounds and minimize the formation of agglomerates that can negatively impact the mechanical response of the device. Due to the ease of processing, light weight, and low costs, these approaches can be promising to fabricate cellulose/silver nanocomposites for different applications, including innovative filters for environmental remediation, conductive films for smart electronics, nanoparticles for a selective colorimetric detection, semiconductor nanocomposites for photocatalytic applications, and antibacterial films for food packaging. In this perspective, recent discoveries on the fabrication of cellulose–silver hybrid materials promise a future implementation of pre-industrial methodologies addressed to their sustainable application for large-scale processing.

## Figures and Tables

**Table 1:** Cellulose/silver nanocomposites: summary of preparation methods, properties, and applications.

Cellulose Source	Preparation Method	Properties	Applications	Ref.
Cellulose	Chemical reduction	Ag/ZnO decorated cellulose nanocomposite	Rapid sterilization and eradication	[82]
	Synthesis of silver nanoparticles-covered three-dimensional cellulose	3D cellulose-Ag scaffold	Tissue engineering and other relevant applications	[142]
	Surface sol−gel method	TiO2/Ag nanosponges containing uniform dispersion of silver nanoparticles	Photocatalysts	[143]
Cellulose fibers	In situ biosynthesis of Ag NPs by sumac leaf extract as reducing and stabilising agent	Face-centered cubic Ag NPs with size of 52 to 105 nm	Ag NP improved the durability of the coating	[83]
Cellulose nanofibers	Thermal treatment and DMF as reducing agents	Good distribution of AgNPs on cellulose nanofibers	Antimicrobial activities	[77]
	Decoration with AgNPs via ultraviolet radiation and copper nanoparticles via chemical reduction	The metal release related to the contents of copper or silver	Superior bactericidal activity	[85]
	Directional freeze-drying	Silver nanowires	Anisotropic 3D composite sponge	[144]
Celluose nanocrystals	Nucleation of silver nanoparticles		Mediators for silver nanoparticles preparation with good size distribution	[43]
Cellulose acetate nanofibers	In situ synthesis of silver nanoparticles followed by electrospinning technique	Dense and compact entangled nanofibers	An efficient anticorrosive material	[92]
Bacterial cellulose	UV light irradiation	AgNPs with narrow size distribution along with some aggregate	Antimicrobial membrane for wound-healing treatment	[20]
	Hydrogel. In situ reduction of Ag NPs	Homogeneous distribution of Ag NPs inside BC hydrogel	Broad-spectrum antimicrobial performance	[87]
	Nanocrystals. Chemical reduction of Ag+ ions	High metallic Ag content ranging from 88% to 97%	Food packaging, paints, or surface treatment	[94]
	Silver nanoparticles ~16.5 nm were thermal reduction	In situ synthesized on TEMPO oxidized bacterial cellulose nanofiber surfaces by	Wound dressing	[145]
Oxidized bacterial cellulose	Ion-exchange followed by thermal reduction	Controlled size distribution		[54]
Dicarboxylic cellulose	In situ immobilization of silver nanoparticles	Uniform silver nanoparticles with 15 nm size.	Dicarboxylic cellulose/silver nanocomposite	[19]
Oxidized cellulose microfibrils containing aldehyde groups	Silver mirror reaction	Particle size ranged from 5 to 25 nm	Materials had an electric conductivity of approximately 5 S/cm	[34]
Dialdehyde nanofobrillated cellulose	In situ immobilization of silver nanoparticles	Silver nanoparticles (~31.07 nm) were fabricated and uniformly anchored	Controlled release and long-term antibacterial	[146]
Hydroxypropyl cellulose.	Silver-coated zinc oxide nanoparticles by solution blending	Multifunctional composite films	Accelerated wound-healing, antibacterial properties	[35]
TEMPO-oxidized cellulose nanofibrils	Silver nanoparticles diameter range of 8−25 nm	In situ reduction to form CNF/silver nanoparticle Suspention	Selective detection of cysteine	[147]
Cellulose ultrathin films grafted by *N*,*N′*-carbonyldiimidazole	In situ immobilization of silver nanoparticles	Higher silver density regions	Enable controlled electrical conductivity of cellulose surfaces	[61]
Cellulose pulp	Hydrothermal in situ reduction followed by dry-jet wet-spun	Homogenous distributed silver among the fiber cross section	Yellow fabrics	[76]
Cellulose paper	The addition of various cellulose derivatives suppresses aggregation of Ag NPs during reduction	The concentration of Ag NPs is proportional to the initial silver salt concentration	Enhanced antibacterial activity of the cotton fibers	[86]
	Dip-coating technique	Silver nanowire	Cellulose/silver nanowires papers	[148]
Filter paper	Silver nanoparticles	Reduction and immobilization	Catalyst for or 4-nitrophenol reduction, and to emphasize its duality as a SERS substrate	[149]
Cellulose nanowhiskers	Chemical reduction	Homogeneous AgNPs	Antimicrobial activity and biomedical applications	[81]
Electrospun cellulose acetate nanofiber	Electrospun nanomats of cellulose acetate with the incorporation of Ag NPs	Green synthesized silver nanoparticles (3–8 nm)	Activity towards biofilms, healthcare, and design of antimicrobial nanomat and wound dressing	[91]
Porous cellulose	Ion exchange of carboxylate groups to Ag cations followed by the reduction	Composite cellulose/Ag particles	Catalysis	[78]
Porous cellulose particles	Solvent-releasing method: silver cation exchange reduction reaction using the carboxylate groups	Composite cellulose/Ag particles	Catalysis	[124]
Cellulose/Keratin	One-Pot Synthesis	27 ± 2 for Ag^0^ and 9 ± 1 nm for Ag^+^	Blends containing either Ag^+^ or Ag^0^	[65]
Regenerated cellulose	Hyperbranched polyamide-amine/silver nanoparticles	In situ	Food packaging	[150]

## Data Availability

Not applicable.

## Supplementary Materials

The following are available online at https://www.mdpi.com/article/10.3390/biom11111684/s1. Figure S1: (a) Cellulose in plants structure to nanometer scale. (b) The schematic diagram of the acid hydrolysis of cellulose to obtain nanocellulose. (c) Synthesized bacterial cellulose nanofibers (Miyashiro, Hamano, & Umemura, 2020). (Reproduced under Creative Commons Attribution, Copyright 2020, Daisuke Miyashiro et al., 2020). Figure S2: TEM of CNF (A) and TEMPO-oxidized CNF (B) isolated from date palm fruit stalks (M. Hassan et al., 2017). (C) Cellulose nanocrystals obtained by sulfuric acid hydrolysis from rice husk (Abou-Zeid et al., 2018), and (D) SEM images of freeze-dried BC harvested after two days (magnification: 50,000×) (E. Hassan, Hassan, Abou-zeid, Berglund, & Oksman, 2017). Reproduced under Creative Commons Attribution, Copyright 2017 Hassan et al. Reproduced under a Creative Commons Attribution 4.0 International License Abou-Zeid et al., 2018. Figure S3: Physical method of preparation of AgNPs–cellulose composites (Xu, Li, Yue, & Lu, 2017). Open access. Figure S4: TEM of (a) AgNPs, (b) CNCs, and (c) silver-loaded nanocellulose (Fan et al., 2020). Figure S5: Schematic illustration of the formation of PVA or poly (nisopropylacrylamide) films with cellulose nano- whiskers functionalized with carboxylate groups/AgNPs (Spagnol et al., 2018). Copyright 2018, with permission from Elsevier. Figure S6: An illustration of the fabrication of cellulose nanofiber mats containing HAp and Ag NPs. In this section, the various steps of fabrication are described sequentially. Figure S7: Schematic illustration of oxidized cellulose/AgNPs beads and its catalytic ability (Fujii, Imagawa, Omura, Suzuki, & Minami, 2020). Figure S8: Role of BC hydrogel groups in the synthesis of AgNPs. Figure S9: Formation of Ag nanorods by the CNC. Figure S10: Schematic illustration of the one-pot fabrication process of CNCs-PVP-Ag nano-hybrids. Figure S11: Reaction process and surface morphology of films: (a) Reaction scheme of Ag–MPTS–cellulose cross-linked structure; (b) reaction process of films; (c,e) SEM graphs of RCF and CANF0.04 surface; (d,f) SEM graphs of RCF and CANF0.04 cross section. Figure S12: Artwork of silver nanoparticles formation on the bacterial cellulose matrix (Pal, Nisi, Stoppa, & Licciulli, 2017). Figure S13: UV-vis spectra of CNF–AgNPs mixed with 20 different amino acid.
