# Supplementary material for: Cellulose–Silver Composites Materials: Preparation and Applications
Source: Biomolecules. 2021 Nov 12;11(11):1684. doi: 10.3390/biom11111684 (PMC8615592; doi:10.3390/biom11111684)
Supplement: Supplementary file 1 [file biomolecules-11-01684-s001.zip › biomolecules-1440141-supplementary.pdf]

biomolecules

Supplementary

Cellulose–Silver Composites Materials: Preparation and Applications

Ahmed Salama [1,*], Ragab E. Abouzeid [1], Medhat E. Owda [2], Iriczalli Cruz-Maya [3] and Vincenzo Guarino [3,*]

1 Cellulose and Paper Department, National Research Centre, 33 El-Bohouth St., Dokki, Giza 12622, Egypt; r_abouzeid2002@yahoo.com
2 Chemistry Department, Faculty of Science, Al-Azhar University, Nasr City, Cairo 11884, Egypt; medhatowda@gmail.com
3 Institute of Polymers, Composite and Biomaterials, National Research Council of Italy, Mostra D'Oltremare, Pad 20, V. J.F. Kennedy 54, 80125 Naples, Italy; cdiriczalli@gmail.com
* Correspondence: ahmed_nigm87@yahoo.com (A.S.); vincenzo.guarino@cnr.it (V.G.); Tel.: +20-1008842629 (A.S.); +39-0812425944 (V.G.)

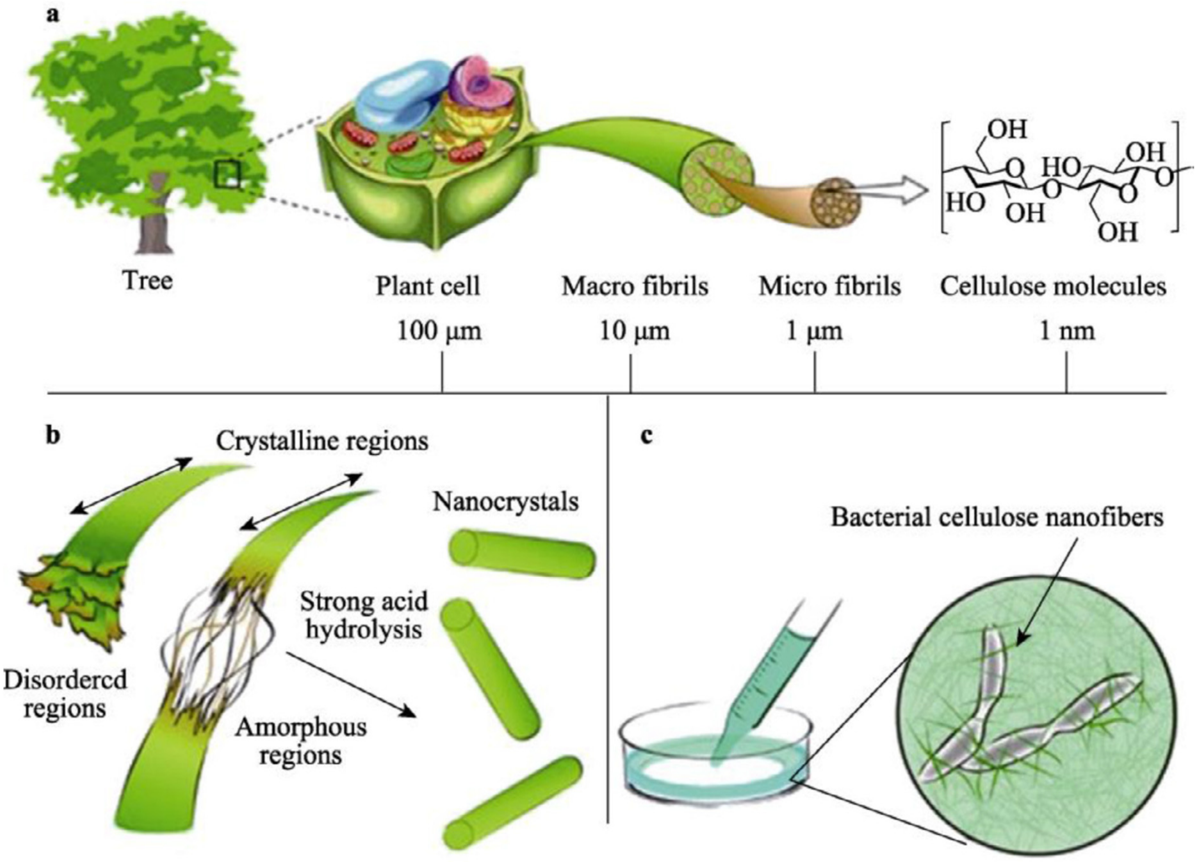

Figure S1. (**a**) Cellulose in plants structure to nanometer scale. (**b**) The schematic diagram of the acid hydrolysis of cellulose to obtain nanocellulose. (**c**) Synthesized bacterial cellulose nanofibers [1]. (Reproduced under Creative Commons Attribution, Copyright 2020, Daisuke Miyashiro et al. 2020).

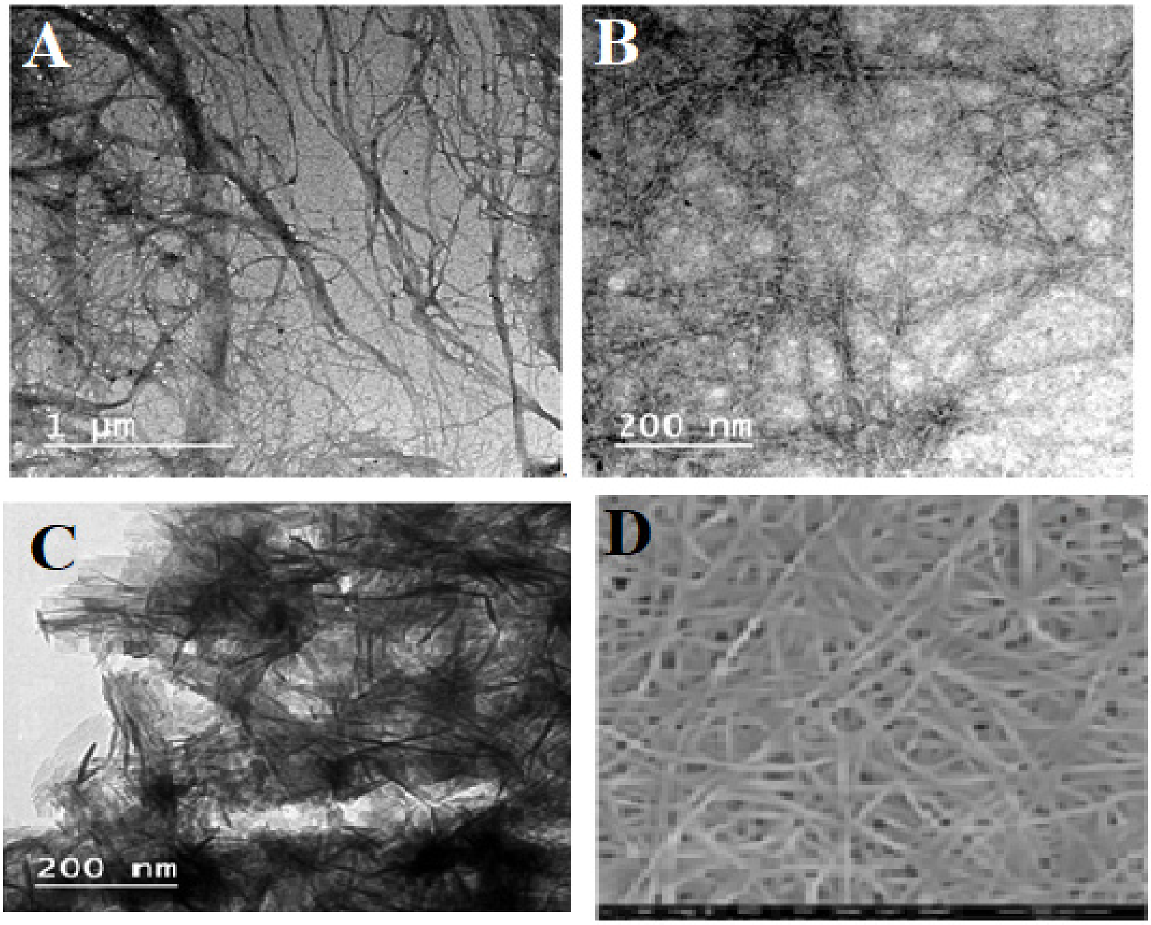

Figure S2. TEM of CNF (**A**) and TEMPO-oxidized CNF (**B**) isolated from date palm fruit stalks (M. Hassan et al., 2017). (**C**) Cellulose nanocrystals obtained by sulfuric acid hydrolysis from rice husk [2], and (**D**) SEM images of freeze-dried BC harvested after two days (magnification: 50,000 X) [3]. Reproduced under Creative Commons Attribution, Copyright 2017 Hassan et al. Reproduced under a Creative Commons Attribution 4.0 International License Abou-Zeid et al., 2018.

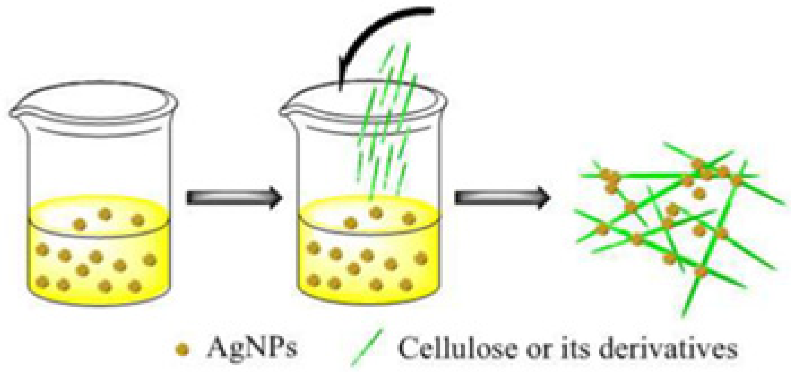

Figure S3. Physical method of preparation of AgNPs-Cellulose composites [4]. Open access.

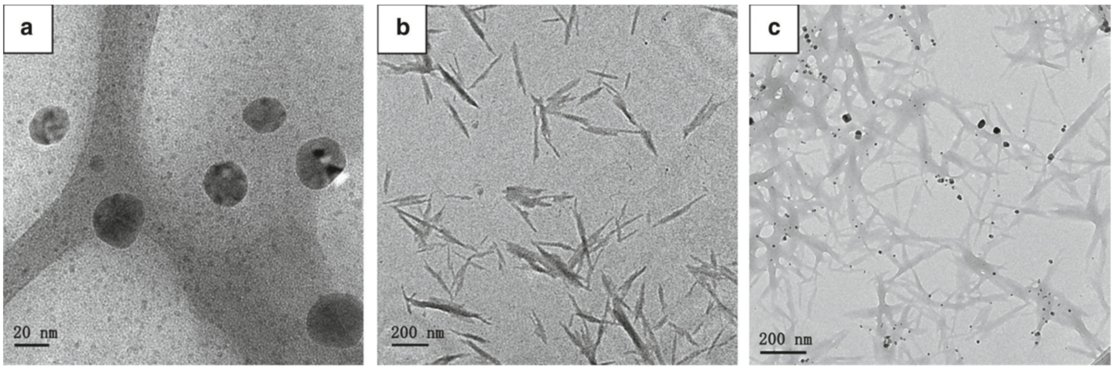

Figure S4. TEM of (**a**) AgNPs, (**b**) CNCs and (**c**) silver-loaded nanocellulose [5].

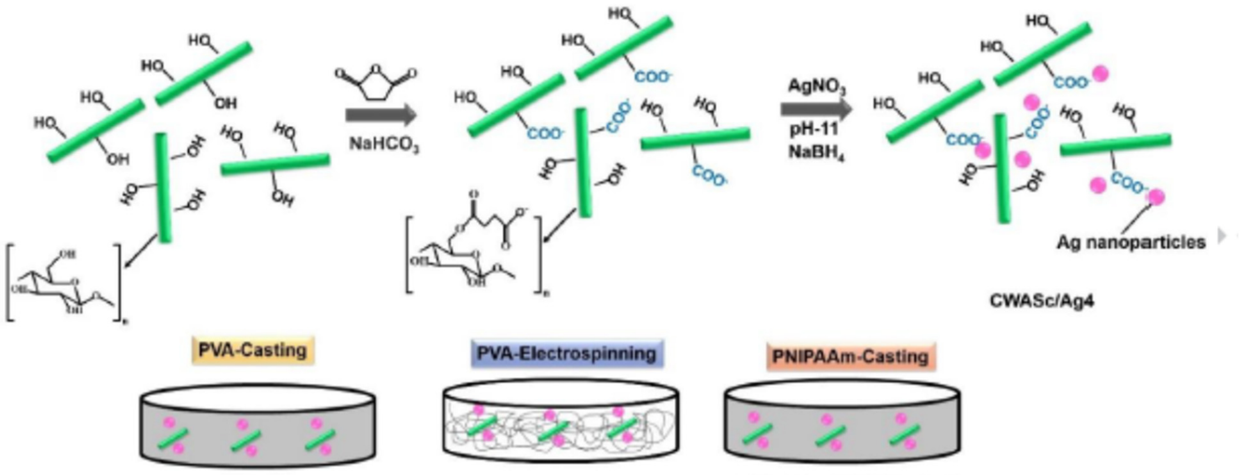

Figure S5. Schematic illustration of the formation of PVA or poly (Nisopropylacrylamide) films with cellulose nanowhiskers functionalized with carboxylate groups/AgNPs (Spagnol et al., 2018). Copyright 2018, with permission from Elsevier.

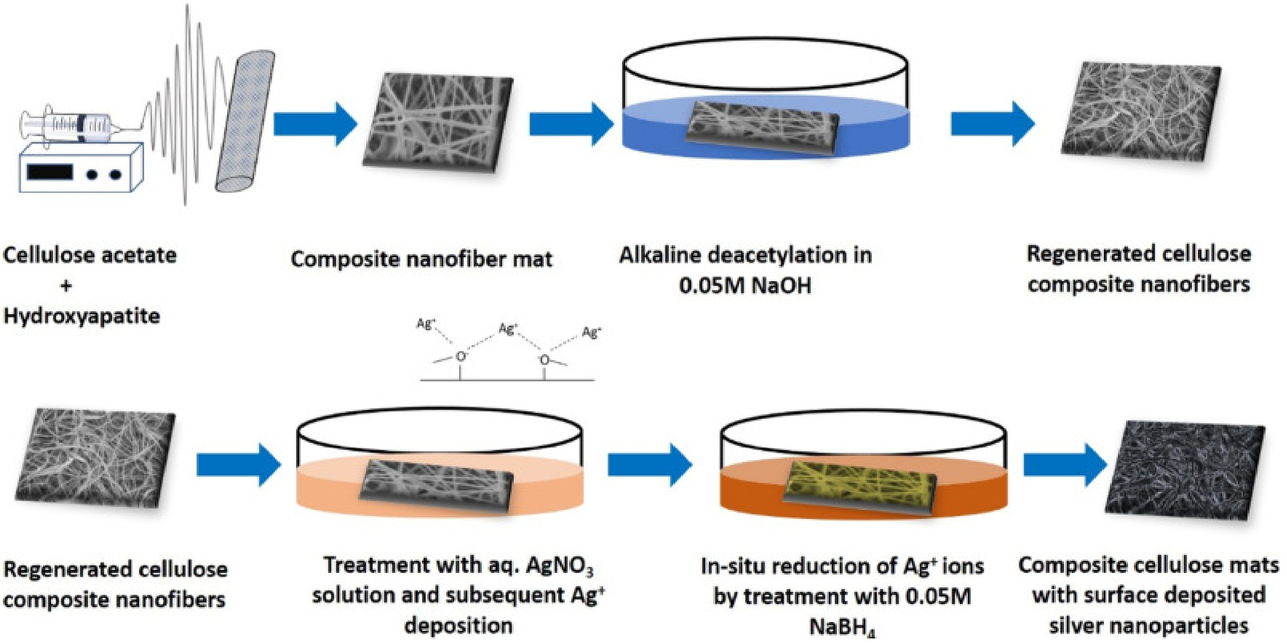

Figure S6. An illustration of the fabrication of cellulose nanofiber mats containing HAp and Ag NPs. In this section, the various steps of fabrication are described sequentially.

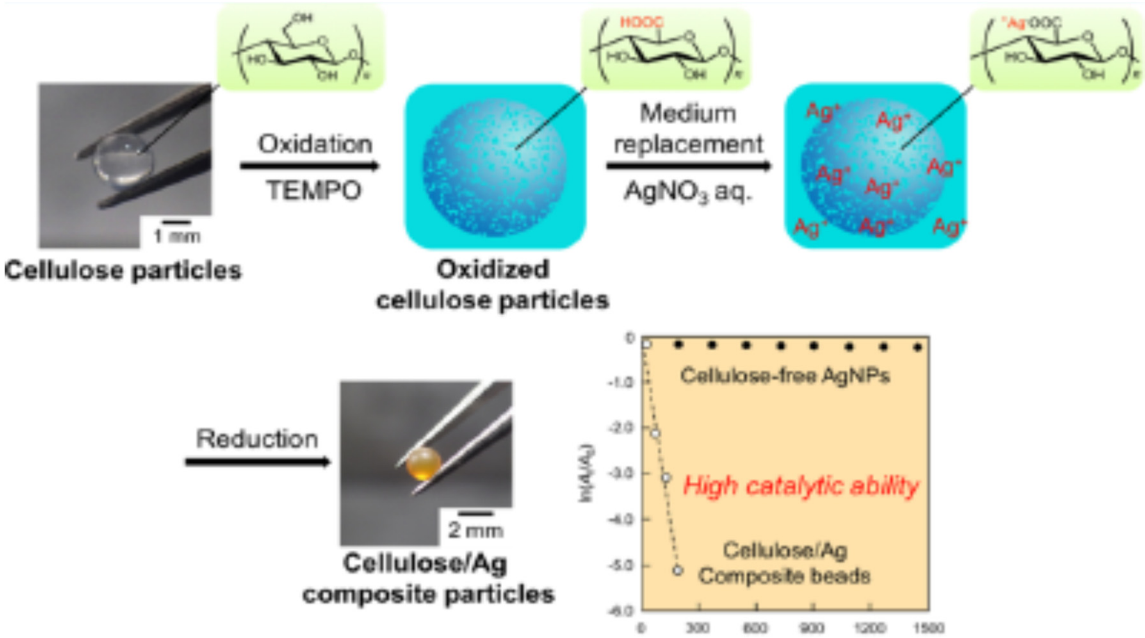

Figure S7. Schematic illustration of oxidized cellulose/AgNPs beads and its catalytic ability [7].

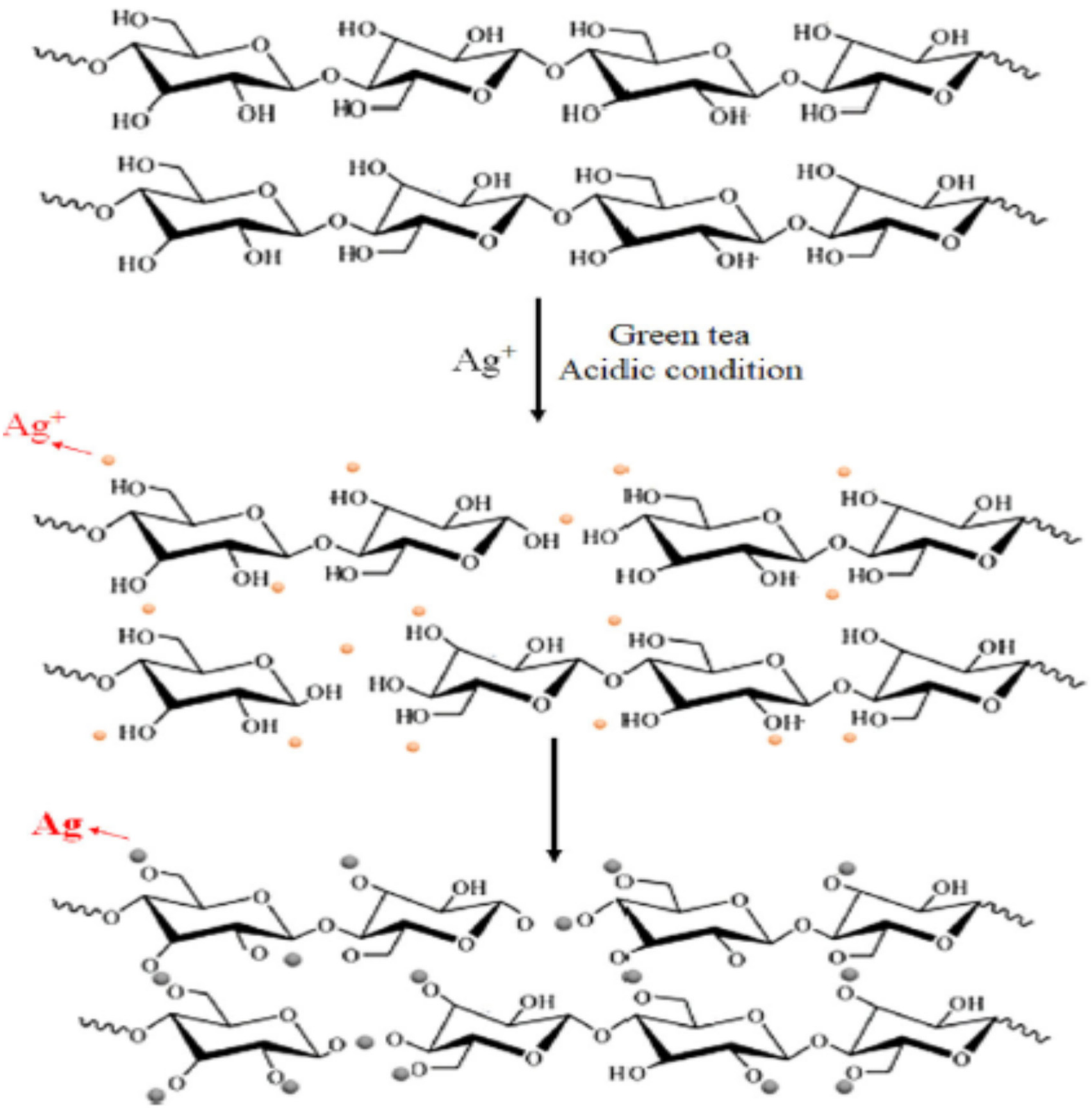

Figure S8. Role of BC hydrogel groups in the synthesis of AgNPs.

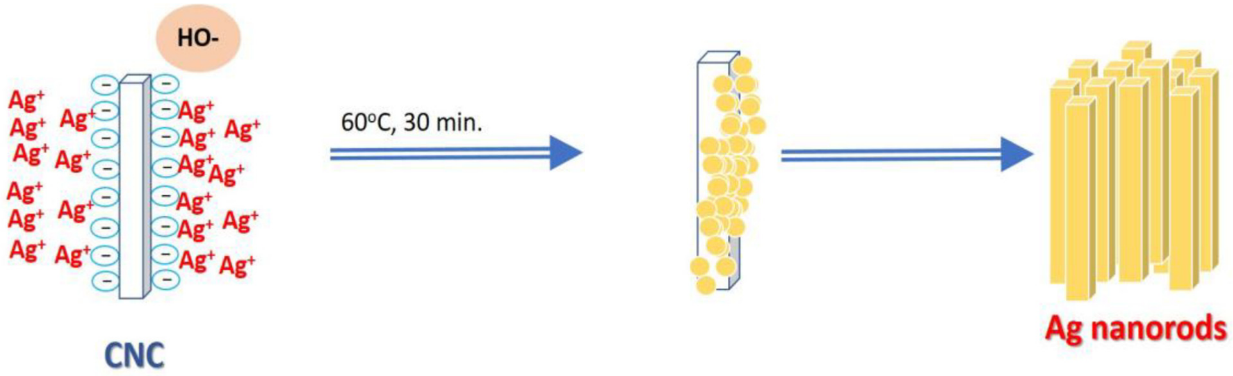

Figure S9. Formation of Ag nanorods by the CNC.

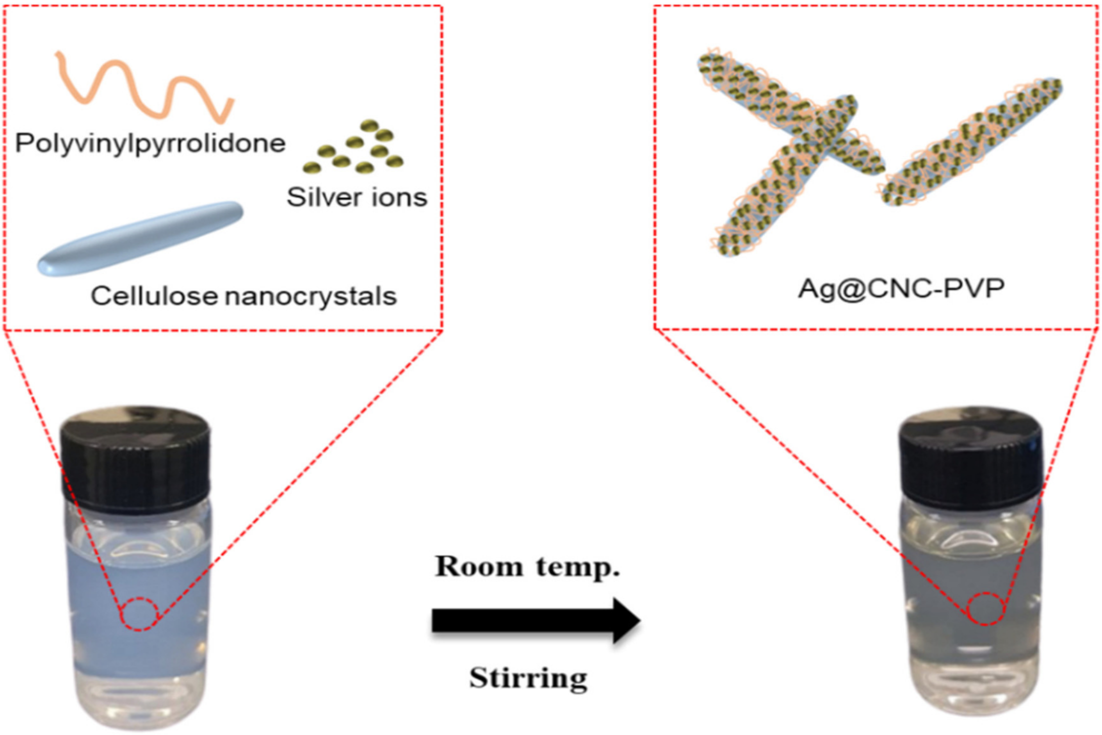

Figure S10. Schematic illustration of the one-pot fabrication process of CNCs-PVP-Ag nanohybrid.

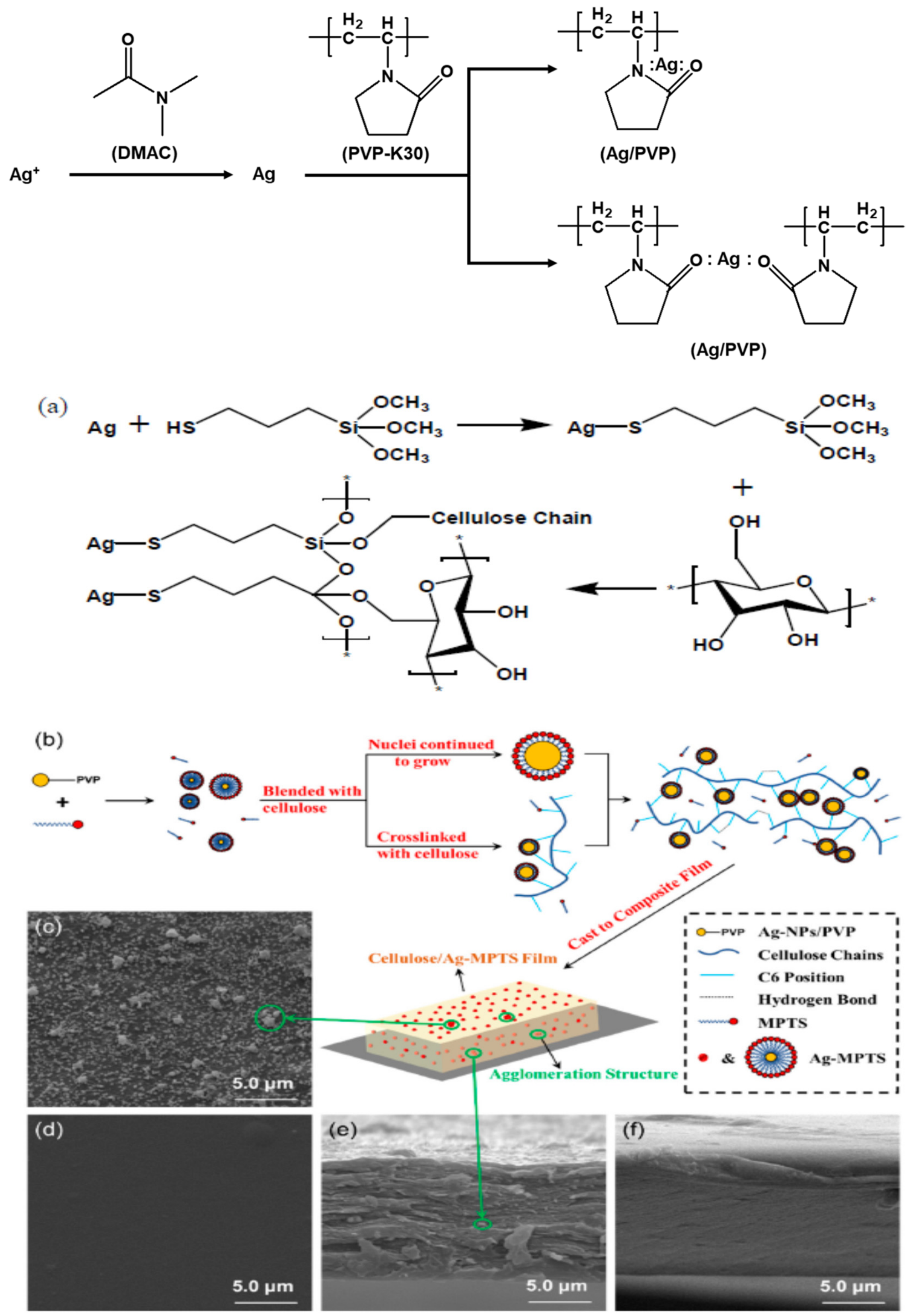

Figure S11. Reaction process and surface morphology of films: (**a**) Reaction scheme of Ag-MPTS-Cellulose cross-linked structure; (**b**) Reaction process of films; (**c**,**e**) SEM graphs of RCF and CANF0.04 surface; (**d**,**f**) SEM graphs of RCF and CANF0.04 cross-section.

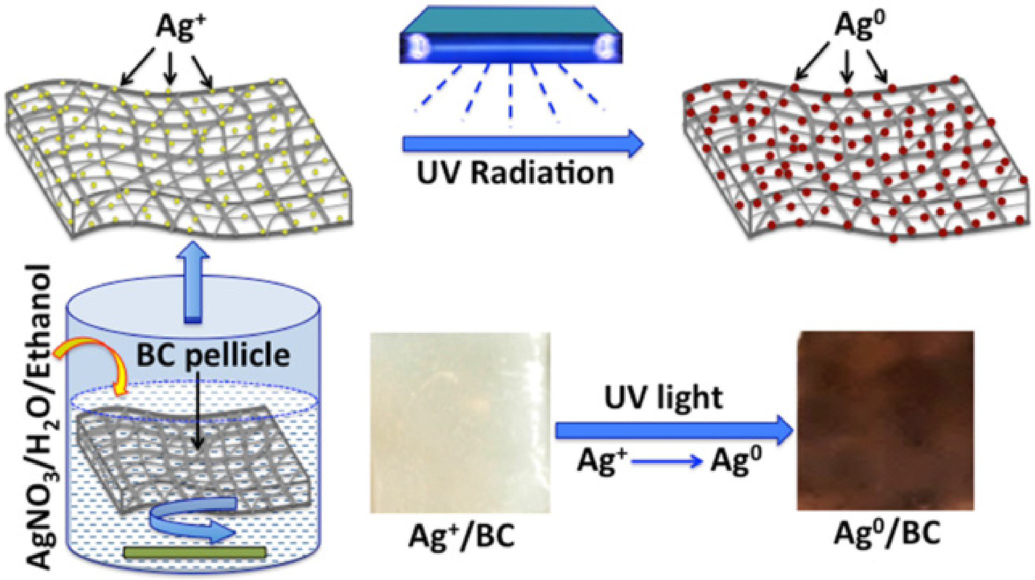

[a]Photographs of pellicles before and after UV irradiation are also presented.

Figure S12. Artwork of silver nanoparticles formation on the bacterial cellulose matrix (Pal, Nisi, Stoppa, & Licciulli, 2017).

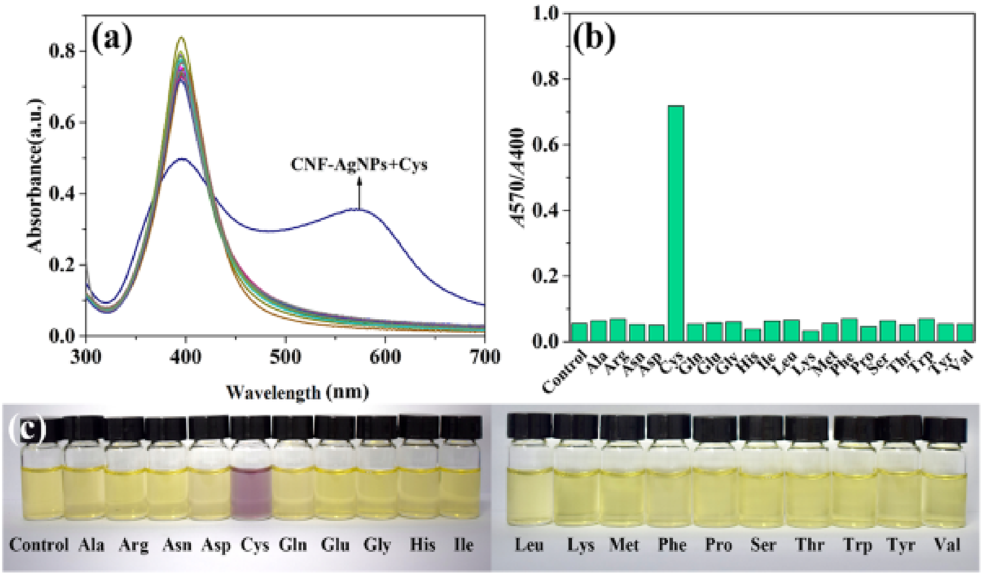

Figure S13. UV-vis spectra of CNF-AgNPs mixed with 20 different amino acid.

References

1. Miyashiro, D., Hamano, R., & Umemura, K. (2020). A Review of Applications Using Mixed Materials of Cellulose, Nanocellulose and Carbon Nanotubes. Nanomaterials, 10(2), 186. https://doi.org/10.3390/nano10020186
2. Abou-Zeid, R. E., Diab, M. A., Mohamed, S. A. A., Salama, A., Aljohani, H. A., & Shoueir, K. R. (2018). Surfactant-Assisted Poly(lactic acid)/Cellulose Nanocrystal Bionanocomposite for Potential Application in Paper Coating. Journal of Renewable Materials, 6(4), 394–401. https://doi.org/10.7569/JRM.2017.634156
3. Hassan, E., Hassan, M., Abou-zeid, R., Berglund, L., & Oksman, K. (2017). Use of Bacterial Cellulose and Crosslinked Cellulose Nanofibers Membranes for Removal of Oil from Oil-in-Water Emulsions. Polymers, 9(12), 388. https://doi.org/10.3390/polym9090388
4. Xu, Y., Li, S., Yue, X., & Lu, W. (2017). Review of Silver Nanoparticles (AgNPs)-Cellulose Antibacterial Composites. BioResources, 13(1). https://doi.org/10.15376/biores.13.1.Xu
5. Fan, L., Zhang, H., Gao, M., Zhang, M., Liu, P., & Liu, X. (2020). Cellulose nanocrystals/silver nanoparticles: in-situ preparation and application in PVA films. Holzforschung, 74(5), 523–528. https://doi.org/10.1515/hf-2018-0251
6. Spagnol, C., Fragal, E. H., Pereira, A. G. B., Nakamura, C. V., Muniz, E. C., Follmann, H. D. M., … Rubira, A. F. (2018). Cellulose nanowhiskers decorated with silver nanoparticles as an additive to antibacterial polymers membranes fabricated by electrospinning. Journal of Colloid and Interface Science, 531, 705–715. https://doi.org/10.1016/j.jcis.2018.07.096
7. Fujii, Y., Imagawa, K., Omura, T., Suzuki, T., & Minami, H. (2020). Preparation of Cellulose/Silver Composite Particles Having a Recyclable Catalytic Property. https://doi.org/10.1021/acsomega.9b03634
8. Pal, S., Nisi, R., Stoppa, M., & Licciulli, A. (2017). Silver-Functionalized Bacterial Cellulose as Antibacterial Membrane for Wound-Healing Applications. ACS Omega, 2(7), 3632–3639. https://doi.org/10.1021/acsomega.7b00442
